# Comparing frailty index distribution, mortality and frailty dynamics across multiple countries: applying the ‘zero-state’ principle to harmonised ageing data

**DOI:** 10.1093/ageing/afag242

**Published:** 2026-08-17

**Authors:** Saskia Drijver-Headley, Judith Godin, Kenneth Rockwood, Peter Hanlon

**Affiliations:** School of Health and Wellbeing, University of Glasgow, Glasgow, United Kingdom of Great Britain and Northern Ireland; Division of Geriatric Medicine, Dalhousie University, Halifax, Canada; Frailty & Elder Care Network, Nova Scotia Health, Halifax, Canada; Division of Geriatric Medicine, Dalhousie University, Halifax, Canada; Frailty & Elder Care Network, Nova Scotia Health, Halifax, Canada; School of Health and Wellbeing, University of Glasgow, Glasgow, United Kingdom of Great Britain and Northern Ireland

**Keywords:** frailty, international comparisons, mortality, ageing trajectories, older people

## Abstract

**Introduction:**

Frailty is a dynamic state linked to disability, hospitalisation and mortality. Cross-country comparisons of frailty progression remain limited. We compared frailty distribution, mortality risk and deficit accumulation across 17 middle−/high-income countries. We then examined whether outcomes among individuals with minimal baseline deficits (‘zero-state’) reflect broader population health patterns to explore utility of the ‘zero-state’ as a marker of population health.

**Methods:**

Data were from five harmonised longitudinal ageing surveys (Health and Retirement Survey, Survey of Health, Ageing and Retirement in Europe, English Longitudinal Study of Ageing, Chinese Health and Retirement Longitudinal Survey, Mexican Health and Ageing Study). A 31-item frailty index (FI) was constructed using identical variables at baseline, 2- and 4-year follow-up. Participants with <2 deficits were classified as in ‘zero-state’. Outcomes: all-cause mortality and FI change among survivors. Logistic regression (mortality) and negative binomial models (deficit accumulation) were adjusted for age, sex and baseline FI. Analyses were stratified by zero-state and non-zero-state.

**Results:**

Baseline frailty varied between countries: highest in the USA, Mexico and Southern/Eastern European nations, and lowest in Northern/Central Europe. Mortality and deficit accumulation differed substantially. Mexico showed highest mortality and fastest accumulation, while Switzerland and Scandinavian countries showed the lowest. Zero-state analyses revealed larger between-country differences, but lower statistical power, than analyses of those with higher FI. Some countries (e.g. USA, UK) showed favourable zero-state outcomes but more rapid progression among individuals with higher baseline FI.

**Conclusion:**

Frailty distribution, mortality and frailty progression vary widely across settings, with poorer outcomes in lower-income countries. Zero-state trajectories capture important population differences but do not fully reflect patterns among those with existing frailty, potentially reflecting within-country inequalities.

## Key Points

- Frailty distribution, mortality and progression vary widely across 17 middle- and high-income countries.
- Lower-income countries show higher frailty, faster deficit accumulation and higher mortality.
- Northern and Central European countries have the lowest frailty levels and slowest progression.
- Zero-state analyses reveal population health differences but may mask within-country inequalities.

## Introduction

Frailty is an age-related loss of the ability to resist potentially damaging stress (i.e. less robustness) or repair it when it occurs (less resilience) [1]. People living with frailty are at increased risk of adverse health outcomes such as falls, hospitalisation, dementia, disability and mortality [2], leading to higher use of healthcare services and healthcare costs [3, 4]. Healthcare systems across the world need to adapt to rising levels of frailty [5].

Comparing frailty between countries provides insights into the population health and needs of older adults [6]. Frailty is multidimensional, as it involves a combination of physical, psychological and functional elements [2, 7]. It is also dynamic, with changes over time within individuals [8, 9]. Understanding variations in frailty between countries can help to understand specific frailty-related challenges within each country’s population. A few studies have compared frailty between countries: mostly prevalence, incidence or using global burden of disease estimates of indicator conditions [10–12]. These studies do not necessarily reflect the changing nature of frailty within individuals. An alternative method is to compare the components of frailty and their dynamics between countries using survey data with repeated measures within individuals and items harmonised between different settings.

Our paper has two broad aims: the first is to compare population-level frailty by assessing the distribution of the frailty index (FI) (a cumulative deficit model of frailty), the rate of change in the FI, and mortality risk and assessing how each of these metrics varies between countries. The second is to explore whether frailty dynamics and mortality risk among the healthiest in the population can serve as a useful metric of the ambient health of the population. It has been hypothesised that the background ambient health of a population may be estimable by assessing those individuals with the fewest health deficits at baseline (so-called ‘zero-state’) [13, 14]. Differences in mortality risk and deficit accumulation among the ‘healthiest’ in society would be expected to reflect environmental or population-level differences [14, 15]. However, it has yet to be demonstrated whether the outcomes of these individuals are reflective of the wider health of the population, and whether the ‘zero-state’ gives an accurate measure of how a nation’s health compares with others’. Therefore, we compare the distribution of frailty, change in frailty over time, and mortality risk between 17 countries to assess how these measures vary between countries and assess whether findings among those in the ‘zero-state’ reflect the health of the wider population.

## Methods

**Figure 1:**
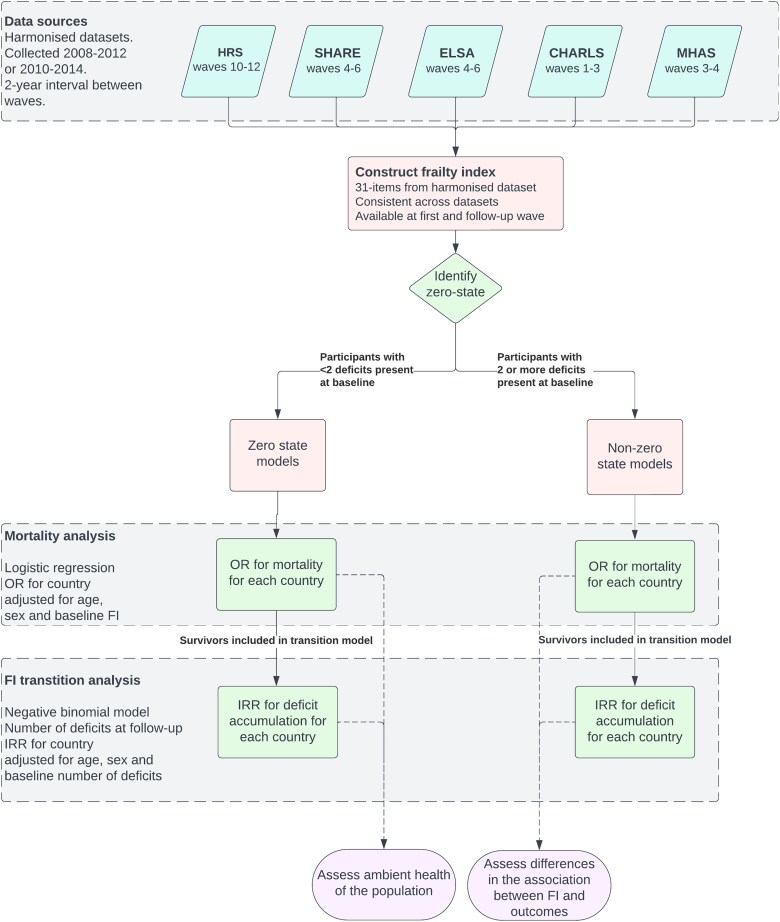
Outline of analysis. CHARLS: Chinese Health, Ageing and Retirement Longitudinal Study, ELSA: English Longitudinal Study of Ageing, HRS: Health and Retirement Survey, IRR: incident rate ratio, MHAS: Mexican Health and Ageing Survey, OR: Odds ratio, SHARE: Survey of Health Ageing and Retirement in Europe, OR: odds ratio, IRR: incidence rate ratio.

An overview of the analyses is shown in Figure 1.

### Data sources

Our data come from five longitudinal surveys of ageing and health: the Health and Retirement Survey (HRS), the Survey of Health, Ageing and Retirement in Europe (SHARE), English Longitudinal Study of Ageing (ELSA), Chinese Health and Retirement Longitudinal Survey (CHARLS), and Mexican Health and Ageing Study (MHAS). We selected waves to maximise contemporaneous data between settings. We used the harmonised versions of these surveys, compiled by the Gateway for Global Ageing, to ensure consistent coding of individual variables to allow cross-country comparison [16]. Each survey comprises repeated waves of data collection from a sample of older people. We selected waves to maximise comparability between countries, in terms of the variables collected, contemporaneous collection of these variables, and the time interval between baseline and follow-up (2- and 4-years for all included surveys). Wave selection is summarised in Figure 1. Within each selected survey, we selected individuals aged 50 years or older.

### FI construction

**Figure 2:**
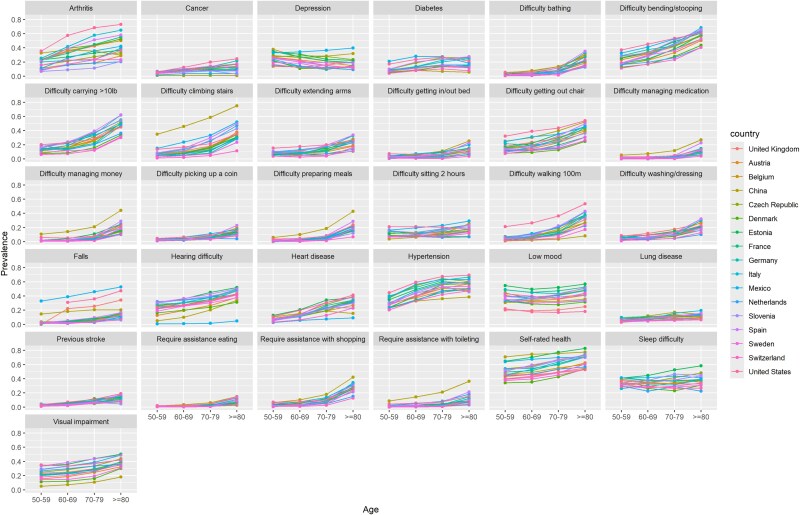
Prevalence of individual FI deficits by age in all included countries.

The FI is a quantitative frailty assessment based on a cumulative deficit model of frailty. The FI is calculated by dividing the sum of health deficits an individual has by the number of deficits that are being assessed [17]. The FI is a score between 0 (no deficits present) and 1 (all deficits present) [18]. The deficits can include symptoms, diseases, abnormal clinical findings and functional deficits. Deficits must be linked to poor health status, increase in prevalence with age, and be neither too rare (<1% prevalence) nor too common among older people (e.g. 80% prevalence by age 80). When calculating FI, at least 30 deficits must be included [19]. Given the focus in this analysis on comparisons between countries, we restricted the included deficits to those that had been harmonised between the different data sources and that had the same questions, definitions and thresholds across each survey and at each follow-up time. Definitions are shown in the Supplementary Appendix 1. We identified 31 deficits fulfilling the criteria for inclusion in the FI, summarised in Figure 2.

### Covariates

In addition to those variables included in the FI, we assessed age (reported at the baseline wave), gender, education (primary, secondary or tertiary), household income and smoking status. For each participant, as reported in each survey.

### Outcomes

Outcomes of interest were all-cause mortality and change in the FI, assessed over 2- and 4-year follow-up. Mortality was assessed using the vital status data in each survey (coded as alive, deceased or unknown). Follow-up FI was assessed (conditional on survival) using data from subsequent survey waves (assessed at 2-year intervals) and using the same list of deficits as used in the baseline FI.

### Missing data

Where data were missing for specific deficits within individuals, the FI was calculated as the number of deficits present divided by the number of deficits with complete data for that individual (i.e. missing deficits were excluded from the numerator and the denominator). Individuals with >20% missing deficits were excluded from the analysis. To minimise bias arising from unequal sampling probabilities and non-response (including wave attrition), all models were estimated using survey sample weights, providing population-representative inference. Countries for which no follow-up data were available at a given time point were excluded from that analysis but assessed at other time points for which data were available.

### Statistical analyses

Each of the analyses described was performed on the full study population, as well as separately with male and female participants.

#### Descriptive statistics

We calculated summary statistics (sample size, mean FI, mean age, percentage of females and the percentage of missing follow-up and missing mortality data) for each country.

#### Distribution of frailty and its components

Histograms and summary statistics (median, interquartile range) were made to visually assess the distribution of the FI in each country overall and stratified by age and sex.

#### Mortality models

We assessed mortality using a logistic regression (as mortality was only available as a binary variable at each wave, precluding time-to-event analyses). We included all countries in the same model using country as an independent. Our main analysis adjusted for age and sex, as our aim was to compare frailty between countries and not to explain the reasons for these differences. In exploratory analyses, we also adjusted for participant education, smoking status and household income.

Separate models were fitted for people in the zero-state and the remainder of the sample for each country giving adjusted odds ratios for mortality for each country along with 95% confidence intervals. The UK was used as the reference category.

#### Frailty transition models

Analyses on change in deficits from baseline to follow-up were carried out on survivors with complete follow-up data. Count-based regression models were used to assess differences in change in FI between countries, adjusting for age, sex and baseline FI (plus education, smoking and income for exploratory analyses). For this, the FI was re-scaled to a count of deficits present, rounded to the nearest whole number (i.e. a count between 0 and 31 deficits). This allowed count-based regression models to be used to model the number of follow-up deficits adjusted for age, sex and number of baseline deficits. Various count-based models were explored for assessing the association between covariates (country, age, sex and baseline deficits) and the number of deficits at follow-up. A negative binomial model was found to fit the follow-up counts the best. Over dispersion was observed when using a Poisson model, reflecting the right-skewed distribution of the FI. We calculated incidence rate ratios treating country as the exposure.

#### Zero-state analyses

For each data source, we identified participants with the lowest baseline FI (zero-state), defined as people with fewer than 2 deficits present. Subsequent analyses were conducted in parallel on participants in the zero-state (representing the ambient health status of the population) and on those with higher degrees of frailty (i.e. 2 or more deficits). Our hypothesis was (i) that the health outcomes of those in the zero-state (such as the probability of mortality of the rate of increase in deficits within the FI) would reflect the background health of the population (and therefore serve as a basis for comparison between different countries) and (ii) that differences between countries when assessing those in the zero-state would be similar to differences of the wider population (differences in the zero-state are hypothesised to reflect the effect of the setting or environment on health without being confounded by individual health status). We report the zero-state differences alongside when estimating each country’s mortality and transition rates.

## Results

The demographic characteristics of the samples from the countries are summarised in Table 1 and stratified by sex in Supplementary Table 4 and Supplementary Table 5. The prevalence of each of the included deficits across age strata is shown in Figure 2 and the distribution of the FI in different countries can be seen in Supplementary Figure 1. All deficits increased with age as expected (although low mood had a slight U-shaped relationship with an increase in people aged 50–59 relative to those aged 60–69). The relative position of different countries varied across deficits. For example, China had the highest proportion of self-reported difficulty climbing stairs, but the lowest proportion of visual impairment. USA had the highest prevalence of self-reported arthritis or difficulty walking 100 metres. The he ordering of countries was consistent between the main analysis and after stratifying by age and after omitting each deficit from the frailty index in turn (Supplementary Figure 3 & 16) and after omitting each deficit from the frailty index in turn.

**Table 1:** Baseline characteristics and completeness of follow-up.

Country	Mean age	Mean FI					*N* complete baseline data	*N* (%) in zero-state at baseline	% missing at baseline	*N* complete follow-up data (2 years)	Deaths (0–2 years)	% missing at 2 years	*N* complete follow-up data (4 years)	Deaths (2–4 years)	% missing at 4 years
		All	Age 50–59	Age 60–69	Age 70–79	Age ≥80									
Austria	65.9	0.14	0.1	0.12	0.17	0.25	4967	1535 (30.9%)	0.8%	3889	173	18.8%	2987	276	34.8%
Belgium	65.3	0.17	0.13	0.15	0.19	0.28	5029	938 (18.7%)	0.4%	3794	180	21.3%	3358	349	26.6%
China	62.5	0.17	0.13	0.17	0.22	0.31	12 882	3013 (23.4%)	1.6%	11 199	400	11.4%	10 678	899	11.5%
Czech Republic	65.7	0.17	0.12	0.15	0.21	0.29	5262	1047 (19.9%)	0.8%	3917	255	21.3%	3488	473	25.3%
Denmark	65	0.12	0.09	0.11	0.15	0.23	2162	716 (33.1%)	0.4%	1864	93	9.9%	1644	172	16.4%
Estonia	66.6	0.2	0.15	0.18	0.24	0.31	6536	778 (11.9%)	0.6%	5280	389	13.8%	4600	729	19.0%
France	66.4	0.16	0.13	0.14	0.18	0.28	5414	948 (17.5%)	1.5%	3790	175	27.8%	3058	294	39.0%
Germany	67.9	0.18	0.14	0.15	0.18	0.3	1609	243 (15.1%)	0.1%	1091	30	30.4%	973	63	35.7%
Italy	66.7	0.17	0.1	0.15	0.21	0.34	3482	746 (21.4%)	0.3%	2550	137	23.1%	2337	248	26.0%
Mexico	65.8	0.19	0.15	0.19	0.23	0.29	13 016	2405 (18.5%)	8.6%	11 499	1318	10.0%	–	–	–
Netherlands	65.8	0.13	0.11	0.12	0.15	0.2	2704	685 (25.3%)	0.3%	2197	64	16.7%	–	–	–
Slovenia	65.6	0.16	0.13	0.14	0.2	0.25	2636	526 (20%)	0.6%	1893	75	25.8%	1822	159	25.3%
Spain	68.1	0.21	0.13	0.17	0.24	0.36	3574	650 (18.2%)	0.4%	2889	218	13.4%	2546	431	17.0%
Sweden	69.6	0.14	0.09	0.12	0.14	0.23	1955	517 (26.4%)	0.3%	1486	91	19.6%	1381	146	22.1%
Switzerland	65.5	0.11	0.09	0.1	0.13	0.2	3574	1128 (31.6%)	0.3%	2801	89	19.4%	2535	178	24.3%
UK	65.8	0.16	0.12	0.15	0.19	0.27	10 640	2785 (26.2%)	0.1%	9116	396	10.7%	8399	728	14.3%
USA	66.9	0.22	0.17	0.2	0.24	0.32	20 565	3154 (15.3%)	0.6%	18 535	1073	5.3%	16 687	2326	8.1%

Follow-up values of the FI among survivors followed a negative binomial distribution conditional on the baseline FI (Supplementary Figure 2), consistent with previous analyses demonstrating that FI values can improve as well as worsen, although the overall population-level trajectory is towards deterioration.

Missing data on the baseline FI was low (<2%) for most countries but higher in Mexico (8.9% missing, 1218 participants, 1211 of which were proxy interviews in which physical function questions were not answered) (Table 1). Proportions of missing follow-up FI and mortality data are summarised in Supplementary Tables 2 and 3 and deficit-level missingness in Supplementary Tables 6–36. Differences in baseline frailty between those retained or lost to follow-up were small.

### Mortality analysis

**Figure 3:**
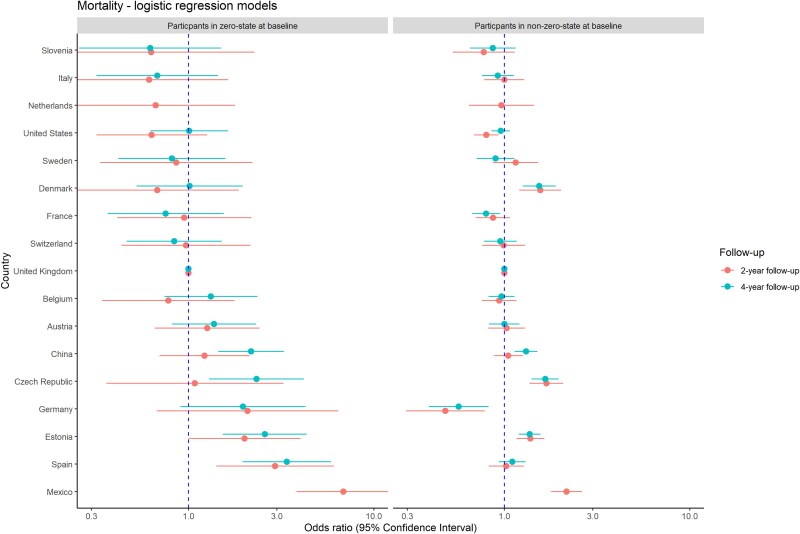
Odds ratios of 2- and 4-year mortality associated with each included country, stratified by zero-state.

The odds ratios for mortality of different countries compared to the UK are shown in Figure 3 for participants in the zero-state (0–1 deficits: left panel) and participants with higher degrees of frailty (2 or more deficits, right panel, adjusted for individual baseline FI-value). Odds of mortality were highest in Mexico for participants in the zero-state and the non-zero-state. In the zero-state analysis, confidence intervals for most of the remaining comparisons included the null apart from Spain and Estonia, which were higher at both time points and China and Czech Republic, which were higher at 4-year follow-up. Among participants in the non-zero-state, the frailty-adjusted odds of mortality were higher in China, Czech Republic and Estonia and possible lower (although with differences between 2- and 4-year follow-up) in France, Sweden, Austria, Germany and Slovenia. In each of these cases, the magnitude of the difference between countries was smaller in the non-zero-state analysis than in the zero-state (i.e. the between-country difference in mortality risk was greater among otherwise healthy people than between those who had some degree of frailty). In exploratory analyses, we found a significant interaction between country and baseline FI with respect to mortality risk. Results were consistent across age and sex strata (Supplementary Figures 4, 5 & 8, 9) and sex (Supplementary Figures 12, 13) strata.

### Transition analysis

**Figure 4:**
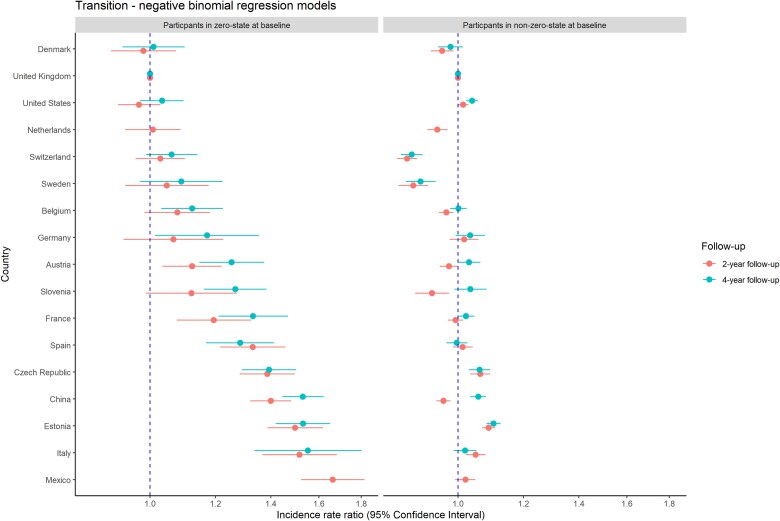
Incidence rate ratios of change in FI deficits at 2- and 4-year mortality associated with each included country, stratified by zero-state.

The rate of deficit accumulation for each country compared to the UK, among participants in the zero-state and those in the non-zero-state, is shown in Figure 4. The highest rate of deficit accumulation was seen in Mexico (both zero- and non-zero-state analyses), followed by some Southern and Eastern European countries (e.g. Estonia, Czech Republic, Italy) and China. This was clearly seen in the zero-state analysis; however, in the analysis of individuals with higher baseline FI, there was some inconsistency across 2- and 4- year follow-up (e.g. for China or Italy, Figure 4, right panel). Rates of deficit accumulation were lowest in Northern and Central European countries such as Switzerland, Sweden, Denmark, the Netherlands and in the UK—most clearly seen in the non-zero-state analysis. The position of the USA with respect to other countries appeared to vary depending on baseline frailty: in the zero-state analysis, deficit accumulation in the USA was comparable to many European nations; however, in the analysis including those with higher FI at baseline, accumulation was more rapid in the USA than in these countries. A similar pattern was also seen with the UK, which had the lowest rate of accumulation in the zero state but, for those with some degree of frailty, rates of accumulation were higher than many Northern/Central European countries. Findings for the transition analysis were similar across age and sex strata (Supplementary Figures 6, 7 & 10, 11).

### Exploratory analyses

Between country differences in mortality and deficit accumulation were similar after additionally adjusting for household income, education and smoking (Supplementary Figures 12–15) and after excluding each individual deficit in turn (Supplementary Figures 17–20).

## Discussion

Using data from large, harmonised longitudinal studies of ageing, we compared the degree and trajectory of frailty between 17 high- and middle-income countries, as well as the mortality risk conditional on baseline frailty. Building upon observations from a large Canadian survey [15], we proposed that mortality risk and frailty progression among the healthiest in each sample (defined as those with the lowest values of the FI: the ‘zero-state’) would reflect the ambient health of the population. Baseline FI was highest in the USA, Mexico and some Southern/Eastern European nations, while baseline FI was lowest in Northern/Central Europe. These differences in frailty distribution reflected large differences in reported prevalence of some components (such as high self-reported hypertension, arthritis and mobility difficulties in the USA). There were substantial differences in mortality risk and in the rate of deficit accumulation between different countries when assessing individuals in the zero-state at baseline. For some countries, these differences were similar in direction (but of smaller magnitude) when comparing individuals at higher degrees of frailty. This suggests that assessing zero-state can pick up important differences between nations’ health, but that the health of people living with frailty can also differ in important ways in some countries and contexts, and that these differences are not reflected in the ‘zero-state’.

Here, some middle-income countries (such as Mexico) and some of the European nations with relatively lower GDP (e.g. Estonia, Czech Republic) exhibit higher average frailty, higher rates of deficit accumulation, and higher mortality risk compared to the higher-income countries. This aligns with the conclusions from two broader meta-analyses and systematic reviews that found higher incidence and prevalence of frailty in LMICs compared to high-income countries [10, 20]. Another study found contrasting results, that higher-income countries have higher levels of frailty than lower-income countries [5]. This might reflect survivor bias, in which people in lower-income countries could be more likely to die at younger ages from other causes and those living to older age may therefore reflect the relatively healthier in society and therefore, at a population level, accumulation of deficits at later life would be less [21]. Our analysis found that Scandinavian countries and Switzerland generally had the lowest average frailty, the lowest rate of deficit accumulation and the lowest mortality risk both in the zero-state and among those with higher baseline FI scores. This finding is consistent with a 2023 study that compared the prevalence of frailty across 15 different SHARE countries, identifying Switzerland and Sweden to have the lowest prevalence of frailty [12, 22]. Another, focusing on 10 SHARE countries, found a higher proportion of frailty in Southern compared to Northern Europe [11].

Given the many factors that contribute to health, assessing national-level health status is challenging. The zero-state hypothesis is that the mortality risk and deficit accumulation among those starting from no deficits (zero-state) is likely to reflect each country’s background health [15]. Instead, we found high statistical uncertainty in the national mortality comparisons owing from the relatively lower sample size and the relatively small number of events in the zero-state groups over 2- or 4-year follow-up. However, for the frailty transition analysis, the precision of estimates was considerably higher. The findings showed the ordering of some countries (particularly the USA, UK, China) differed when comparing the zero state to the non-zero state (those with higher starting frailty). There are several possible explanations for this. Perhaps the differences between the healthiest (zero-state) and those with higher degrees of frailty also differ between countries, meaning that relying on the zero-state as an indicator of a nation’s overall health can obscure differences in health that reflect inequalities within specific countries.

Within countries, poorer health is strongly correlated to lower socio-economic position [23], reflected in differences between zero- and non-zero-state participants in education and household income. However, in exploratory analyses we found that differences in these individual-level factors did little to attenuate between-country differences in deficit accumulation or mortality. To explain the observed between-country differences, future studies should consider wider structural factors including economic and healthcare policies and access, environmental factors, social security and occupational/retirement patterns. We would speculate that these are likely to influence both between-country differences and also the difference between healthier and less healthy people within the same country (e.g. our observation that in the USA people in the zero-state had comparable deficit accumulation to Northern European countries, while those with higher baseline FI scores in the USA experienced more rapid deficit accumulation than people in these countries).

This study used data from large surveys that are nationally representative in terms of age and sex. However, despite the large sample overall, the small number of participants in the zero-state resulted in low statistical power to detect differences between countries in this group, particularly in the mortality analysis. Like any survey, it relied on the voluntary recruitment of participants, meaning some groups may be underrepresented due to factors such as language barriers. To account for this, we incorporated sampling probability weights in all adjusted models. A strength of this study is that it used a standard method of measuring frailty, which is well validated. For this study, when choosing which deficits were included in the FI calculation, this was limited to deficits which were measured across all data sets. This approach maximises the comparability of the FI between different settings. However, this also means that factors such as cognition were not included as they were not consistently assessed across all surveys and also limits the breadth of deficits included in the index. This could result in the FI not reflecting cognitive dimensions of vulnerability. There is also a broader risk that people with cognitive impairment—who are likely to have higher overall frailty—may also be more likely to be lost to follow-up. However, the availability of proxy interviews in these datasets (in which functional deficits were well covered in all countries except Mexico) partially mitigates this risk. The data sets used for this project had been harmonised by the US National Institutes of Health Gateway to Global Ageing Data platform, meaning that the definition and coding of the individual deficits was consistent across each country. We acknowledge, however, that the included deficits are self-reported and cultural or contextual factors could influence reporting differently in different contexts (e.g. we observed higher missing data for ‘difficulty preparing meals’ among men in Mexico than in any other dataset). Furthermore, by limiting our analysis to nations with contemporaneous and harmonised data available, we were unable to include more lower- and middle-income countries in the analysis. Finally, selecting time points where there was the widest contemporaneous data between countries means that the selected waves were relatively older than some more recent analyses in individual countries.

Frailty trajectories and outcomes vary substantially between countries, with consistently poorer outcomes in lower-income settings, highlighting the influence of social, economic and healthcare contexts. As ageing populations drive rising frailty, especially in LMICs, there is an urgent need for research and policies targeting modifiable individual and structural determinants [23–25]. Investment in prevention and management of frailty is essential to mitigate growing pressures on health systems.

## Supplementary Material

Supplementary_materials_afag242
