# Supplementary material for: Comparing frailty index distribution, mortality and frailty dynamics across multiple countries: applying the ‘zero-state’ principle to harmonised ageing data
Source: Age Ageing. 2026 Aug 17;55(8):afag242. doi: 10.1093/ageing/afag242 (PMC13480471; doi:10.1093/ageing/afag242)

Supplementary appendix for "Comparing frailty distribution, mortality and frailty dynamics across multiple countries: Applying the 'zero-state' principle to harmonised ageing data"

Contents

Included deficits and their definitions 3
Additional baseline descriptive results........ 6
Age stratified results 12
Sex stratified results........ 17
Adjustment for additional individual-level variables........ 19
Deficit-level missing data 21

Supplementary tables

Table 1 Definitions and variable names for included deficits 3
Table 2: Baseline frailty index comparison of participants with and without missing follow-up data........ 8
Table 3 Baseline age and sex comparison of participants with and without missing follow-up data........ 9
Table 4 Baseline characteristics – male participants........ 10
Table 5 Baseline characteristics – Female participants 11
Table 6 Missing data: Difficulty raising arms above head 21
Table 7 Missing data: Arthritis 21
Table 8 Difficulty bathing 22
Table 9 Missing data: Difficulty getting in/out of bed........ 22
Table 10 Missing data: Hypertension........ 23
Table 11 Missing data: Cancer 24
Table 12 Missing data: Difficulty carrying 10 pounds or more........ 24
Table 13 Missing data: Difficulty rising from a chair........ 25
Table 14 Missing data: Difficulty picking up a coin........ 25
Table 15 Missing data: Depression 26
Table 16 Missing data: Diabetes........ 26
Table 17 Missing data: Difficulty dressing 27
Table 18 Missing data: Difficulty eating........ 28
Table 19 Missing data: Fallen in the past 2 years 28
Table 20 Missing data: Hearing difficulty........ 29
Table 21 Missing data: Heart problems........ 29
Table 22 Missing data: Lung problems........ 30
Table 23 Missing data: Difficulty preparing meals 31
Table 24 Missing data: Difficulty managing medication 31
Table 25 Missing data: Difficulty managing money 32
Table 26 Missing data: Feels sad 32
Table 27 Missing data: Difficulty with shopping........ 33
Table 28 Missing data: Difficulty sitting for 2 hours........ 34
Table 29 Missing data: Sleep problems........ 34
Table 30 Missing data: Self-rated health 35
Table 31 Missing data: Difficulty climbing stairs........ 35

Table 32 Missing data: Difficulty stooping 36
Table 33 Missing data: Stroke 36
Table 34 Missing data: Difficulty toileting 37
Table 35 Missing data: Visual problems 38
Table 36 Missing data: Difficulty walking 100 metres 38

Supplementary figures

Figure 1 Frailty index distribution by country 6
Figure 2 Number of deficits at 2-year follow-up stratified by baseline deficits 7
Figure 3 Median (interquartile range) frailty index stratified by age group 12
Figure 4 Age stratified mortality analyses: zero-state population 13
Figure 5 Age stratified mortality analyses: non-zero-state population 14
Figure 6 Age stratified analyses of accumulation of FI deficits: zero-state population 15
Figure 7 Age stratified analyses of accumulation of FI deficits: non-zero-state population 16
Figure 8 Mortality analysis by sex, 2-year follow-up 17
Figure 9 Mortality analysis by sex, 4-year follow-up 17
Figure 10 Accumulation of FI deficits by sex, 2-year follow-up 18
Figure 11 Accumulation of FI deficits by sex, 4-year follow-up 18
Figure 12 Mortality analysis before and after additional adjustment, 2-year follow-up 19
Figure 13 Mortality analysis before and after additional adjustment, 4-year follow-up 19
Figure 14 Accumulation of FI deficits before and after additional adjustment, 2-year follow-up 20
Figure 15 Accumulation of FI deficits before and after additional adjustment, 4-year follow-up 20
Figure 16 Median (interquartile range) frailty index for each country after excluding each deficit from the frailty index in turn 40
Figure 17 Mortality analysis - 2-year follow-up - zero-state population - after excluding each deficit in turn 41
Figure 18 Mortality analysis - 2-year follow-up - non-zero-state population - after excluding each deficit in turn 42
Figure 19 Deficit accumulation analysis - 2-year follow-up - zero-state population - after excluding each deficit in turn 43
Figure 20 Deficit accumulation analysis - 2-year follow-up - non-zero-state population - after excluding each deficit in turn 44

Included deficits and their definitions

Table 1 Definitions and variable names for included deficits

Deficit	Question + variable name	Coding
Self-rated health	rXshlt: Self-report of health<br><br>All harmonized datasets	Poor = 1,<br>Fair = 0.75<br>Good = 0.5<br>Very good = 0.25<br>Excellent = 0
Difficulty bathing	rXbatha: Some difficulty-Bathing or showering<br><br>All harmonized datasets	Yes = 1, No = 0
Difficulty dressing	rXdressa: Some difficulty-Dressing<br><br>All harmonized datasets	Yes = 1, No = 0
Difficulty rising from a chair	rXchaira: Some difficulty-Getting up from chair<br><br>All harmonized datasets	Yes = 1, No = 0
Difficulty eating	rXeata: Some difficulty-Eating<br>All harmonized datasets	Yes = 1, No = 0
Difficulty raising arms above head	rXarmsa: Some difficulty-Reach/extending arms up<br><br>All harmonized datasets	Yes = 1, No = 0
Difficulty toileting	rXtoilta: Some difficulty-Using the toilet<br><br>All harmonized datasets	Yes = 1, No = 0
Difficulty climbing a flight of stairs	rXclim1a: Some difficulty-Climbing one flight stairs<br><br>All harmonized datasets	Yes = 1, No = 0
Difficulty carrying 10 pounds of weight	rXlifta: Some difficulty-Lift/carrying 5 kgs<br><br>All harmonized datasets	Yes = 1, No = 0
Difficulty with shopping	rXshopa: Some difficulty-Shopping for groceries<br><br>All harmonized datasets	Yes = 1, No = 0
Difficulty preparing meals	rXmealsa: Some difficulty-Preparing hot meals<br><br>All harmonized datasets	Yes = 1, No = 0
Difficulty managing medication	rXmedsa: Some difficulty-Taking medications<br><br>All harmonized datasets	Yes = 1, No = 0
Difficulty managing money	rXmoneya: Some difficulty-Managing money	Yes = 1, No = 0

	All harmonized datasets	
Difficulty walking 100 metres	rXwalk1a: Some difficulty-Walking one block<br><br>All harmonized datasets	Yes = 1, No = 0
Difficulty getting in/out of bed	rXbeda: Some difficulty-Getting in/out of bed<br><br>All harmonized datasets	Yes = 1, No = 0
Difficulty sitting for 2 hours	rXsita: Some difficulty-Sitting for 2 hours<br><br>All harmonized datasets	Yes = 1, No = 0
Difficulty stooping	rXstoopa: Some difficulty-Stoop/kneel/crouching<br><br>All harmonized datasets	Yes = 1, No = 0
Difficulty picking up a coin	rXdimea: Some difficulty-Picking up a coin<br><br>All harmonized datasets	Yes = 1, No = 0
Low mood	rXsad: (HRS, ELSA, CHARLS and MHAS harmonized datasets) - CESD – Felt sad<br><br>mh002_ (SHARE): Sad or depressed last month	Yes = 1, No = 0
Depression	rXdepres: (HRS, ELSA, CHARLS and MHAS harmonized datasets) CESD – Felt depressed<br><br>mh018_ (SHARE): Depression ever	Yes = 1, No = 0
Sleep problems	rXsleepr: (HRS, ELSA, CHARLS and MHAS harmonized datasets) CESD – Sleep was restless<br><br>mh007_ (SHARE): Trouble sleeping	Yes = 1, No = 0
Stroke	rXstroke: Respondent ever had stroke<br><br>All harmonized datasets	Yes = 1, No = 0
Heart problems	rXhearte: Respondent ever had heart problems<br><br>All harmonized datasets	Yes = 1, No = 0
High blood pressure	rXhibpe: Respondent ever had high blood pressure<br><br>All harmonized datasets	Yes = 1, No = 0
Cancer	rXcancre: Respondent ever had cancer<br><br>All harmonized datasets	Yes = 1, No = 0
Diabetes	rXdiabe: Respondent ever had diabetes	Yes = 1, No = 0

	All harmonized datasets	
Arthritis	rXarthre: Respondent ever had arthritis<br><br>All harmonized datasets	Yes = 1, No = 0
Chronic lung disease	rXlunge: Respondent ever had lung disease<br><br>All harmonized datasets	Yes = 1, No = 0
Fall in the past 2 years	Last 2 years, has respondent fallen down<br><br>HRS: rXfall<br>SHARE: ph010d7<br>ELSA: hefla<br>MHAS: c37_12<br>CHARLS: da023	Yes = 1, No = 0
Hearing difficulties	Hearing<br><br>HRS: rXhearing<br>SHARE: ph046_<br>ELSA: hehear<br><br>Or<br><br>Respondent uses hearing/auditory device<br><br>MHAS: c43_12:<br>CHARLS: da005_	Poor = 1,<br>Fair = 0.5,<br>Good, very good or excellent = 0<br><br>Yes = 1,<br>No = 0
Vision difficulties	Respondent's vision with glasses<br><br>HRS: rXsight<br>SHARE: ph046_<br>ELSA: heeye<br>MHAS: c42_12<br>CHARLS: da005_3_	Legally blind or poor = 1,<br>Fair = 0.5,<br>Good, very good or excellent = 0
In variables from harmonized datasets, the X denotes the wave, e.g. for rXshlt (self-rated health), r1shlt is the self-rated health variable for wave 1.		

Additional baseline descriptive results

Figure 1 Frailty index distribution by country

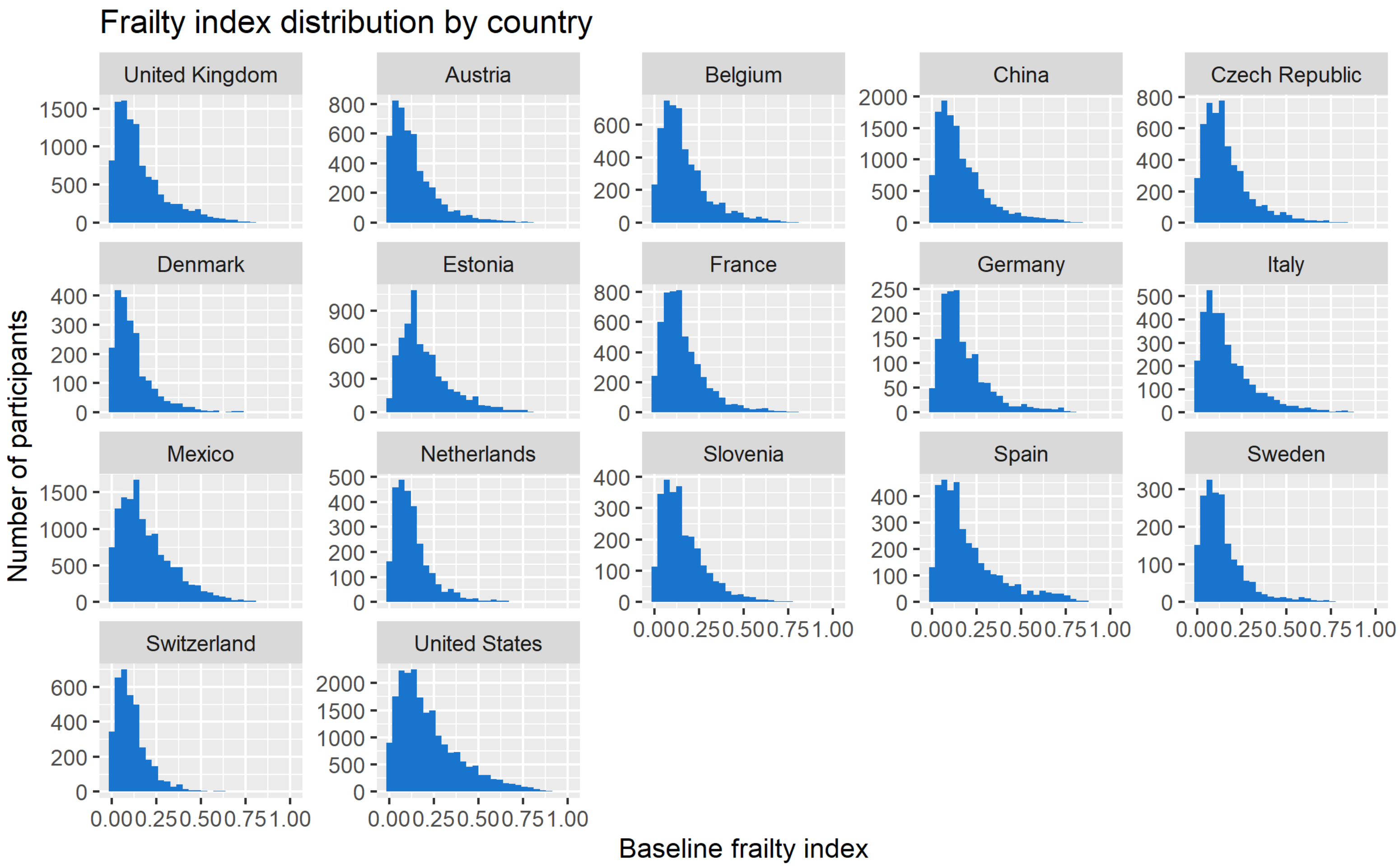

Figure 2 Number of deficits at 2-year follow-up stratified by baseline deficits

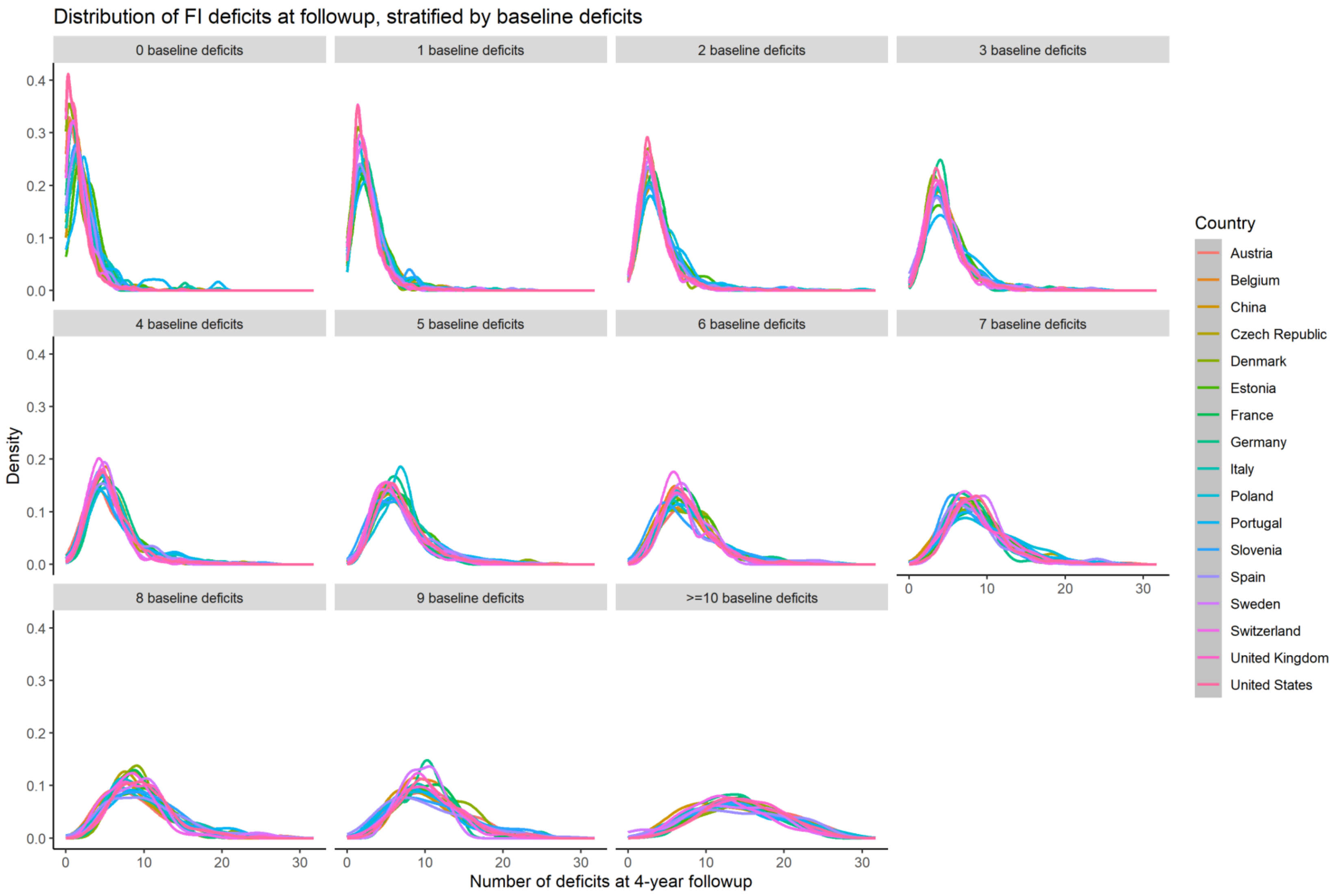

Table 2: Baseline frailty index comparison of participants with and without missing follow-up data.

Country	2-year follow-up: frailty index			4-year follow-up: frailty index			2-year follow-up: mortality			4-year follow-up: mortality		
	Mean baseline FI: Complete follow-up	Mean baseline FI: Missing follow-up	P-value for difference in FI	Mean baseline FI: Complete follow-up	Mean baseline FI: Missing follow-up	P-value for difference in FI	Mean baseline FI: Complete mortality	Mean baseline FI: Missing mortality	P-value for difference in FI	Mean baseline FI: Complete mortality	Mean baseline FI: Missing mortality	P-value for difference in FI
Austria	0.13 (0.12)	0.15 (0.13)	<0.001	0.13 (0.12)	0.13 (0.11)	<0.001	0.14 (0.13)	0.13 (0.13)	0.29	0.14 (0.13)	0.13 (0.13)	0.076
Belgium	0.16 (0.12)	0.16 (0.14)	<0.001	0.15 (0.12)	0.16 (0.13)	<0.001	0.17 (0.13)	0.18 (0.15)	0.19	0.17 (0.13)	0.17 (0.14)	0.75
Czech Republic	0.16 (0.12)	0.16 (0.14)	<0.001	0.15 (0.12)	0.15 (0.13)	<0.001	0.17 (0.13)	0.16 (0.14)	0.12	0.17 (0.13)	0.16 (0.14)	0.017
Switzerland	0.11 (0.09)	0.12 (0.1)	<0.001	0.11 (0.08)	0.11 (0.1)	<0.001	0.11 (0.09)	0.13 (0.11)	0.12	0.11 (0.09)	0.12 (0.1)	0.14
Germany	0.16 (0.12)	0.23 (0.18)	<0.001	0.16 (0.11)	0.21 (0.16)	<0.001	0.17 (0.13)	0.19 (0.15)	0.047	0.17 (0.13)	0.19 (0.16)	0.0021
Denmark	0.12 (0.11)	0.12 (0.11)	<0.001	0.11 (0.1)	0.1 (0.07)	<0.001	0.12 (0.11)	0.13 (0.13)	0.61	0.12 (0.11)	0.13 (0.13)	0.58
Estonia	0.2 (0.14)	0.18 (0.13)	<0.001	0.19 (0.13)	0.18 (0.13)	<0.001	0.2 (0.15)	0.26 (0.19)	0.0024	0.2 (0.15)	0.22 (0.16)	0.12
Spain	0.2 (0.17)	0.17 (0.14)	<0.001	0.19 (0.15)	0.17 (0.14)	<0.001	0.2 (0.18)	0.22 (0.22)	0.38	0.21 (0.18)	0.18 (0.18)	0.004
France	0.16 (0.12)	0.16 (0.13)	<0.001	0.15 (0.11)	0.15 (0.12)	<0.001	0.16 (0.13)	0.16 (0.13)	0.91	0.16 (0.13)	0.16 (0.13)	0.22
Italy	0.17 (0.14)	0.14 (0.13)	0.0022	0.16 (0.13)	0.14 (0.12)	<0.001	0.18 (0.15)	0.16 (0.16)	0.17	0.18 (0.15)	0.16 (0.15)	0.018
Netherlands	0.13 (0.1)	0.14 (0.12)	<0.001	NA	NA	NA	0.13 (0.1)	0.14 (0.12)	0.18	NA	0.13 (0.11)	NA
Sweden	0.13 (0.11)	0.14 (0.13)	<0.001	0.13 (0.11)	0.15 (0.13)	<0.001	0.14 (0.12)	0.16 (0.15)	0.55	0.14 (0.13)	0.14 (0.13)	0.82
Slovenia	0.16 (0.12)	0.15 (0.11)	0.57	0.16 (0.12)	0.15 (0.12)	0.013	0.16 (0.13)	0.18 (0.12)	0.45	0.16 (0.13)	0.15 (0.11)	0.47
United States	0.21 (0.16)	0.2 (0.17)	<0.001	0.2 (0.16)	0.19 (0.15)	<0.001	0.22 (0.17)	0.61 (NA)	NA	0.22 (0.17)	0.21 (0.19)	0.51
United Kingdom	0.15 (0.14)	0.18 (0.16)	<0.001	0.15 (0.13)	0.16 (0.14)	<0.001	0.16 (0.15)	0.15 (0.14)	0.029	0.16 (0.14)	0.17 (0.15)	0.012
Mexico	0.19 (0.14)	0.21 (0.17)	<0.001	NA	NA	NA	0.2 (0.15)	0.16 (0.15)	<0.001	NA	0.19 (0.15)	NA
China	0.16 (0.14)	0.15 (0.13)	<0.001	0.16 (0.13)	0.14 (0.13)	<0.001	0.17 (0.14)	0.18 (0.18)	0.15	0.17 (0.14)	0.16 (0.15)	0.055

Table 3 Baseline age and sex comparison of participants with and without missing follow-up data.

Country	Mean age at baseline (sd)								Percent female							
	2-year follow-up: frailty index		4-year follow-up: frailty index		2-year follow-up: mortality		4-year follow-up: mortality		2-year follow-up: frailty index		4-year follow-up: frailty index		2-year follow-up: mortality		4-year follow-up: mortality	
	Complete	Missing	Complete	Missing	Complete	Missing	Complete	Missing	Complete	Missing	Complete	Missing	Complete	Missing	Complete	Missing
United Kingdom	65.4 (9.4)	66.8 (10.4)	64.9 (9)	63.9 (8.8)	66 (9.7)	64 (9.4)	65.9 (9.7)	65.7 (10.1)	55.10%	51.80%	55%	58%	54.60%	56.30%	54.60%	55%
Austria	65.6 (9.4)	66.2 (9.6)	65.6 (9.2)	66 (9.7)	66 (9.6)	64.6 (9.4)	66.3 (9.6)	65 (9.7)	57.20%	60%	57.70%	65.70%	57.50%	55.40%	57.80%	56.20%
Belgium	65.3 (10.1)	63 (10.2)	64.8 (9.7)	63 (9.9)	65.4 (10.5)	64.4 (10.9)	65.6 (10.5)	64.1 (10.6)	55.30%	52.80%	55.90%	53.90%	54.50%	53.30%	54.80%	52.80%
China	62.3 (8.1)	61.4 (8.5)	61.9 (7.8)	60.9 (8.5)	62.5 (8.3)	63.4 (9.3)	62.5 (8.3)	63.1 (9.1)	50.30%	50.90%	50.70%	50.20%	50.10%	49.50%	50.10%	50.30%
Czech Republic	65.4 (8.6)	64.8 (9.3)	65 (8.4)	65.3 (9.2)	65.9 (9.1)	64.1 (9.8)	66 (9.1)	64.2 (9.5)	58.70%	58.90%	59.80%	58.20%	58.10%	53.10%	58.30%	54.90%
Denmark	64.5 (9.9)	62.8 (9.5)	63.9 (9.4)	63.2 (9.6)	65.1 (10.4)	63.4 (12)	65.1 (10.3)	64.2 (11.5)	54%	49.50%	54.10%	50%	53.70%	51.90%	53.70%	53.40%
Estonia	66.3 (9.4)	64.3 (9.7)	65.9 (9.2)	64 (9)	66.6 (9.7)	69.4 (11.9)	66.6 (9.7)	66.6 (10.8)	60.80%	57.20%	62.10%	56.40%	59.50%	51.60%	59.50%	55.50%
France	66 (10.2)	65.6 (10.8)	65.7 (10)	65.4 (10.5)	66.4 (10.6)	66.2 (10.6)	66.5 (10.6)	65.9 (10.6)	56.70%	55.10%	57.10%	55.10%	56.40%	54.90%	56.50%	55.30%
Germany	66.9 (8.2)	69.4 (10.6)	66.5 (8)	68.6 (9)	67.3 (8.5)	69.6 (9.2)	67 (8.3)	69.7 (9.4)	52.70%	54.30%	52.80%	55.60%	52.70%	53.70%	52.30%	54.20%
Italy	66.5 (8.9)	64.7 (8.9)	66.1 (8.7)	64.1 (8.4)	66.8 (9.3)	66.2 (10.3)	66.9 (9.3)	66.1 (9.9)	54.80%	56.50%	55.20%	62%	54.40%	53.60%	54.80%	52.30%
Mexico	64.6 (8.9)	67.2 (11.5)	NA	NA	65.3 (9.5)	62.8 (9.4)	NA	65.2 (9.5)	57.50%	54.80%	NA	NA	56.90%	50.90%	NA	56.80%
Netherlands	65.6 (9.1)	65 (9.4)	NA	NA	65.8 (9.3)	66.1 (10.7)	NA	65.8 (9.5)	56.20%	47.50%	NA	NA	55.20%	55.80%	NA	55.30%
Slovenia	65.7 (9.8)	63.9 (9.4)	65.2 (9.3)	64 (9.8)	65.5 (9.8)	66.5 (13)	65.5 (9.8)	66.6 (12.6)	57%	55%	58.40%	52.90%	56.10%	51.10%	56.20%	47.60%
Spain	67.8 (10.3)	65 (9.7)	67.2 (9.9)	65.4 (9.5)	68.1 (10.6)	65.3 (11.3)	68.5 (10.6)	63.9 (10.2)	55.20%	54.50%	56%	56.10%	54.60%	50.90%	54.70%	51.70%
Sweden	69 (8.5)	69.3 (9.4)	68.9 (8.4)	68.2 (8.7)	69.6 (9)	66.5 (9)	69.7 (9.1)	67.4 (8.3)	54.60%	54.50%	56%	53.80%	54.30%	43.80%	54.90%	38.80%
Switzerland	65.1 (9.5)	64.3 (10.1)	64.9 (9.3)	64.1 (10)	65.4 (9.9)	67.4 (10.7)	65.5 (9.9)	65.3 (10.1)	54.10%	55.60%	54.30%	56.50%	54.10%	50.90%	54.20%	51.30%
United States	66.4 (10.8)	64.9 (11.2)	65.7 (10.4)	64.5 (10.6)	67 (11.2)	53 (NA)	67 (11.2)	66.2 (10)	57.60%	54.20%	58.10%	54.60%	57.10%	100%	57.10%	57.30%

Table 4 Baseline characteristics – male participants

Country	Mean age	Mean frailty index					N complete baseline data	% missing at baseline	N complete follow-up data (2 years)	Deaths (0-2 years)	% missing at 2 years	N complete follow-up data (4years)	Deaths (2-4 years)	% missing at 4 years
		All	Age 50-59	Age 60-69	Age 70-79	Age ≥80								
Austria	65.6	0.12	0.1	0.11	0.14	0.22	2117	1.20%	1638	77	19.90%	1264	136	34.60%
Belgium	65	0.14	0.11	0.13	0.17	0.24	2291	0.60%	1695	100	22.10%	1480	191	27.50%
China	62.5	0.14	0.11	0.15	0.19	0.27	6230	4.40%	5453	225	12.90%	5097	511	13.90%
Czech Republic	65.5	0.15	0.11	0.13	0.17	0.25	2225	1.10%	1615	135	22.20%	1397	254	26.60%
Denmark	64.7	0.11	0.08	0.1	0.14	0.2	1002	0.30%	858	43	10.30%	754	82	16.80%
Estonia	65.9	0.18	0.13	0.17	0.22	0.27	2658	0.70%	2065	211	14.90%	1742	388	20.40%
France	65.7	0.14	0.11	0.12	0.16	0.25	2373	1.40%	1642	80	28.50%	1313	138	39.70%
Germany	68.6	0.16	0.13	0.14	0.17	0.26	756	0.10%	516	15	29.90%	459	36	34.60%
Italy	67.3	0.15	0.08	0.13	0.18	0.27	1581	0.90%	1150	80	22.90%	1047	139	25.70%
Mexico	66.2	0.16	0.11	0.15	0.19	0.25	5609	10%	4949	628	10.50%	-	-	-
Netherlands	66.2	0.11	0.09	0.1	0.13	0.17	1209	0.40%	963	40	17.40%	-	-	-
Slovenia	65.2	0.15	0.12	0.13	0.19	0.22	1159	0.90%	813	45	26.60%	759	91	27.30%
Spain	67.9	0.17	0.1	0.14	0.19	0.29	1627	0.40%	1293	118	13.60%	1119	231	17.40%
Sweden	70	0.13	0.08	0.11	0.13	0.21	896	0.20%	674	47	19.70%	608	78	23.60%
Switzerland	65.4	0.1	0.08	0.1	0.12	0.16	1646	0.50%	1284	51	19.30%	1156	98	24.20%
United Kingdom	65.7	0.15	0.1	0.14	0.17	0.24	4823	0.20%	4098	212	10.80%	3777	374	14.10%
United States	66.5	0.2	0.15	0.18	0.23	0.29	8794	0.90%	7850	515	5.80%	6983	1118	8.70%

Table 5 Baseline characteristics – Female participants

Country	Mean age	Mean frailty index					N complete baseline data	% missing at baseline	N complete follow-up data (2 years)	Deaths (0-2 years)	% missing at 2 years	N complete follow-up data (4years)	Deaths (2-4 years)	% missing at 4 years
		All	Age 50-59	Age 60-69	Age 70-79	Age ≥80								
Austria	66.2	0.15	0.1	0.13	0.18	0.27	2842	0.70%	2192	96	20.10%	1718	140	35.10%
Belgium	65.6	0.19	0.14	0.17	0.21	0.31	2736	0.40%	2096	80	20.80%	1875	158	26%
China	62.6	0.19	0.15	0.19	0.23	0.29	6231	5.20%	5532	175	13.10%	5218	388	14.70%
Czech Republic	65.7	0.18	0.13	0.16	0.23	0.31	3023	1%	2301	120	20.80%	2089	219	24.50%
Denmark	65.3	0.13	0.1	0.12	0.15	0.24	1160	0.50%	1006	50	9.40%	889	90	16%
Estonia	67.1	0.22	0.16	0.19	0.26	0.32	3877	0.60%	3210	178	13.10%	2857	341	18%
France	66.9	0.18	0.14	0.15	0.2	0.29	3041	1.50%	2148	95	27.40%	1745	156	38.40%
Germany	67.2	0.19	0.14	0.16	0.2	0.33	851	0.20%	575	15	30.80%	514	27	36.60%
Italy	66.2	0.2	0.12	0.16	0.25	0.39	1877	1%	1396	57	23.40%	1288	109	26.30%
Mexico	65.5	0.22	0.18	0.22	0.26	0.33	7372	7.90%	6545	690	9.60%	-	-	-
Netherlands	65.5	0.15	0.12	0.13	0.17	0.23	1495	0.30%	1234	24	16.10%	-	-	-
Slovenia	65.8	0.18	0.13	0.15	0.21	0.27	1476	0.50%	1080	30	25.20%	1063	68	23.80%
Spain	68.2	0.24	0.15	0.19	0.28	0.4	1946	0.40%	1595	100	13.20%	1427	200	16.70%
Sweden	69.2	0.15	0.1	0.13	0.15	0.26	1059	0.40%	812	44	19.50%	773	68	20.90%
Switzerland	65.5	0.12	0.1	0.11	0.14	0.22	1926	0.30%	1516	38	19.50%	1376	80	24.60%
United Kingdom	65.9	0.18	0.12	0.16	0.21	0.3	5817	0%	5018	184	10.60%	4622	354	14.50%
United States	67.3	0.24	0.19	0.22	0.25	0.34	11723	0.80%	10662	558	5.10%	9678	1208	7.90%

Age stratified results

Figure 3 Median (interquartile range) frailty index stratified by age group

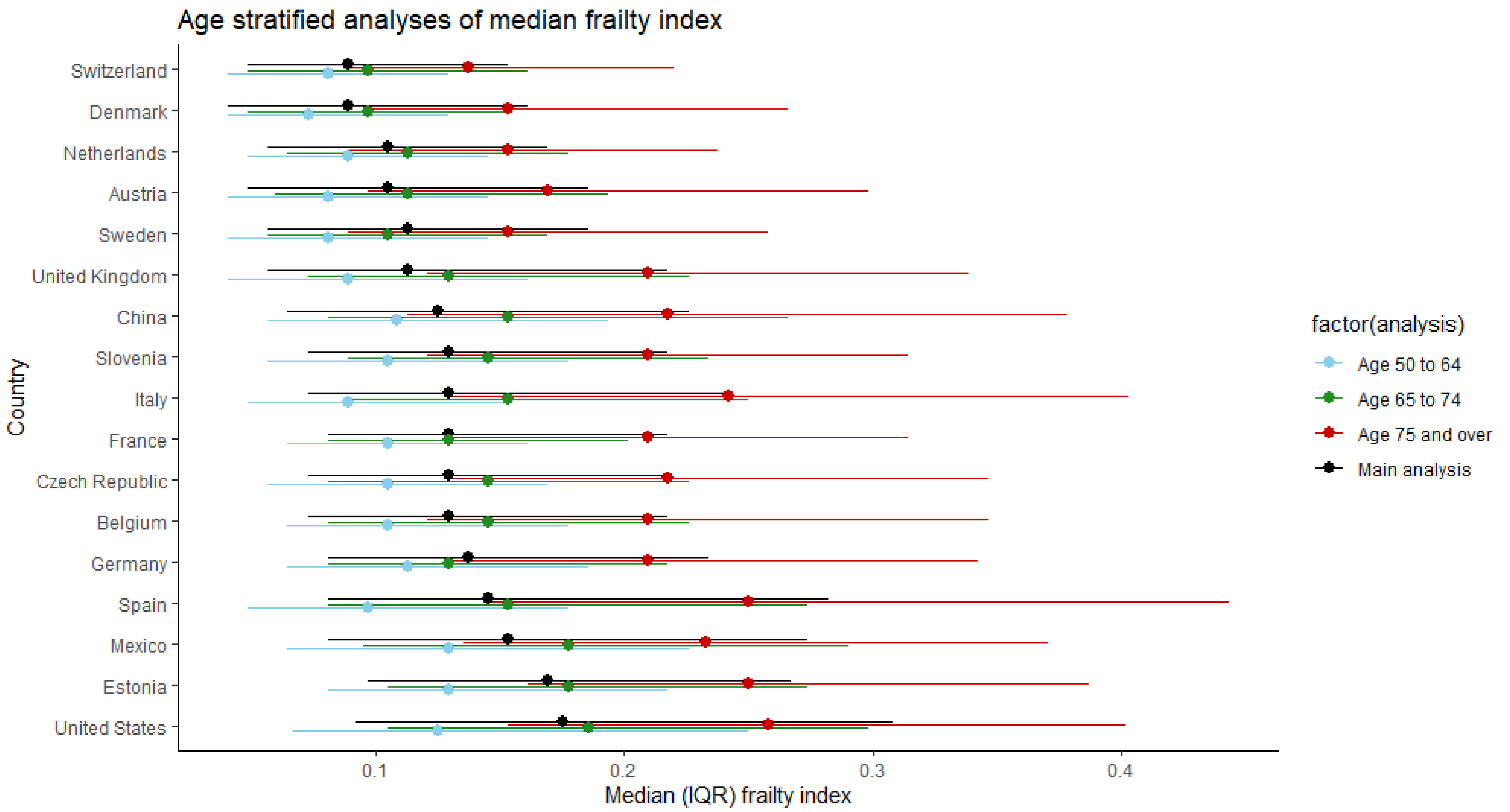

Figure 4 Age stratified mortality analyses: zero-state population

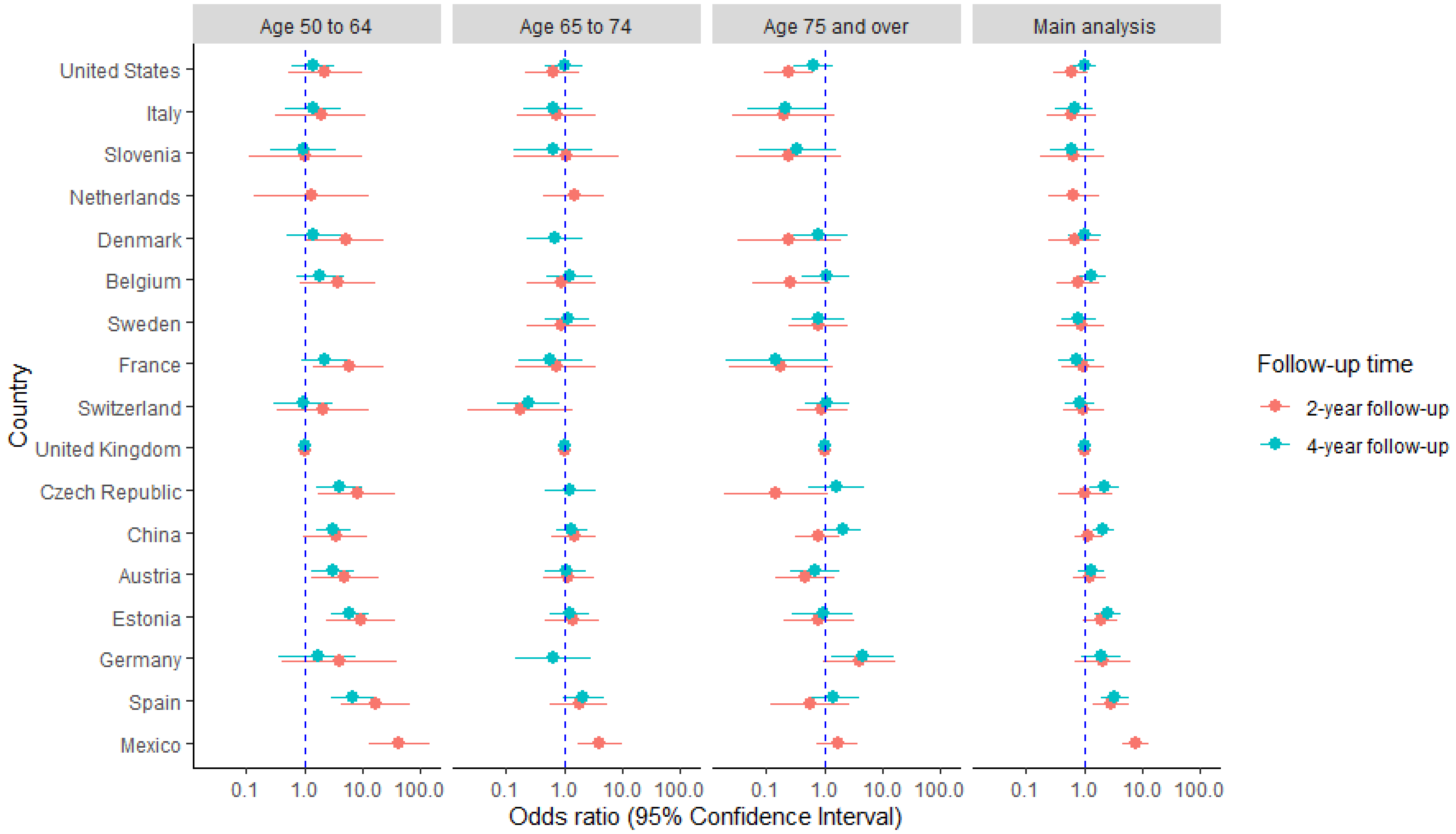

Figure 5 Age stratified mortality analyses: non-zero-state population

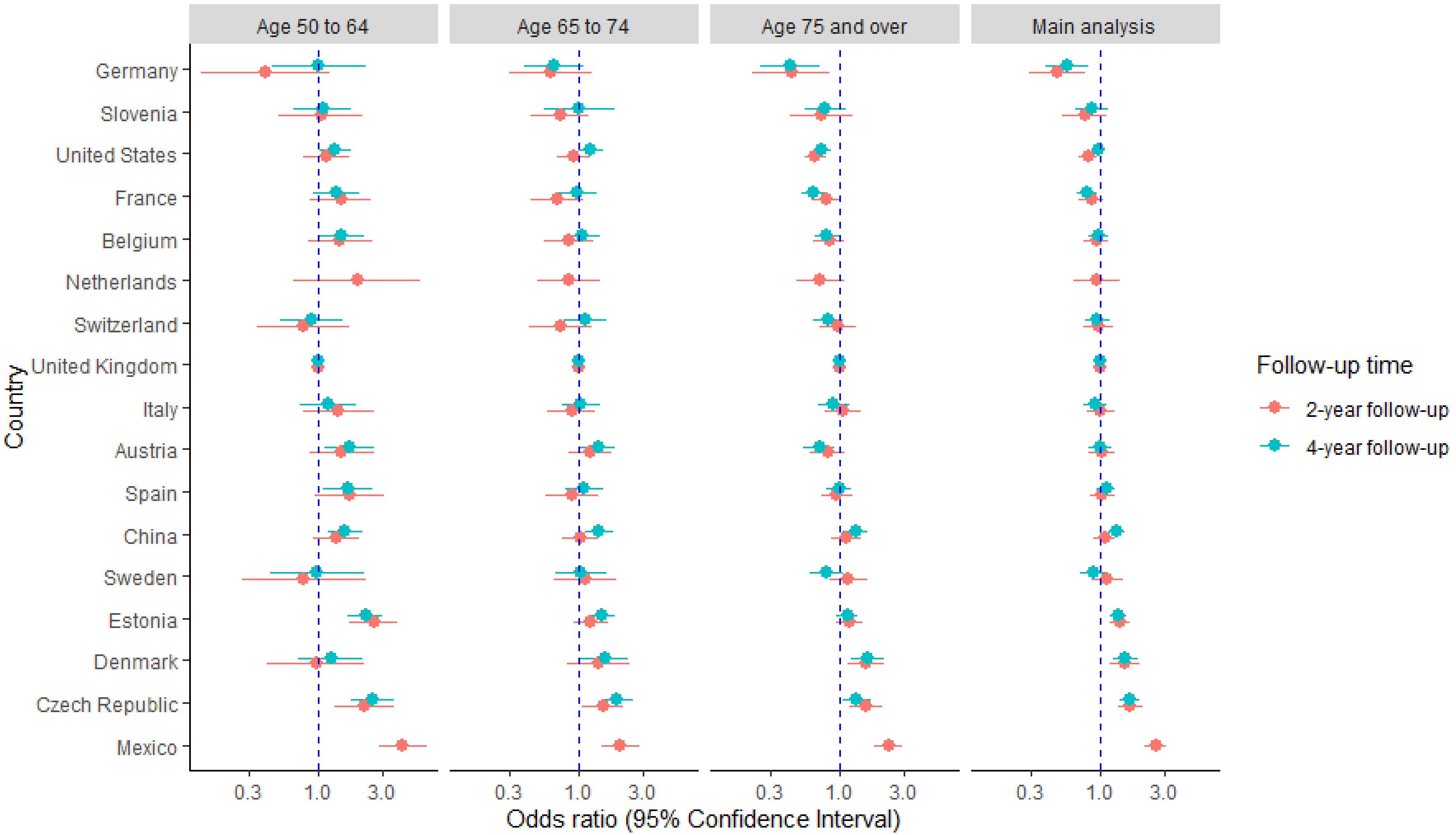

Figure 6 Age stratified analyses of accumulation of FI deficits: zero-state population

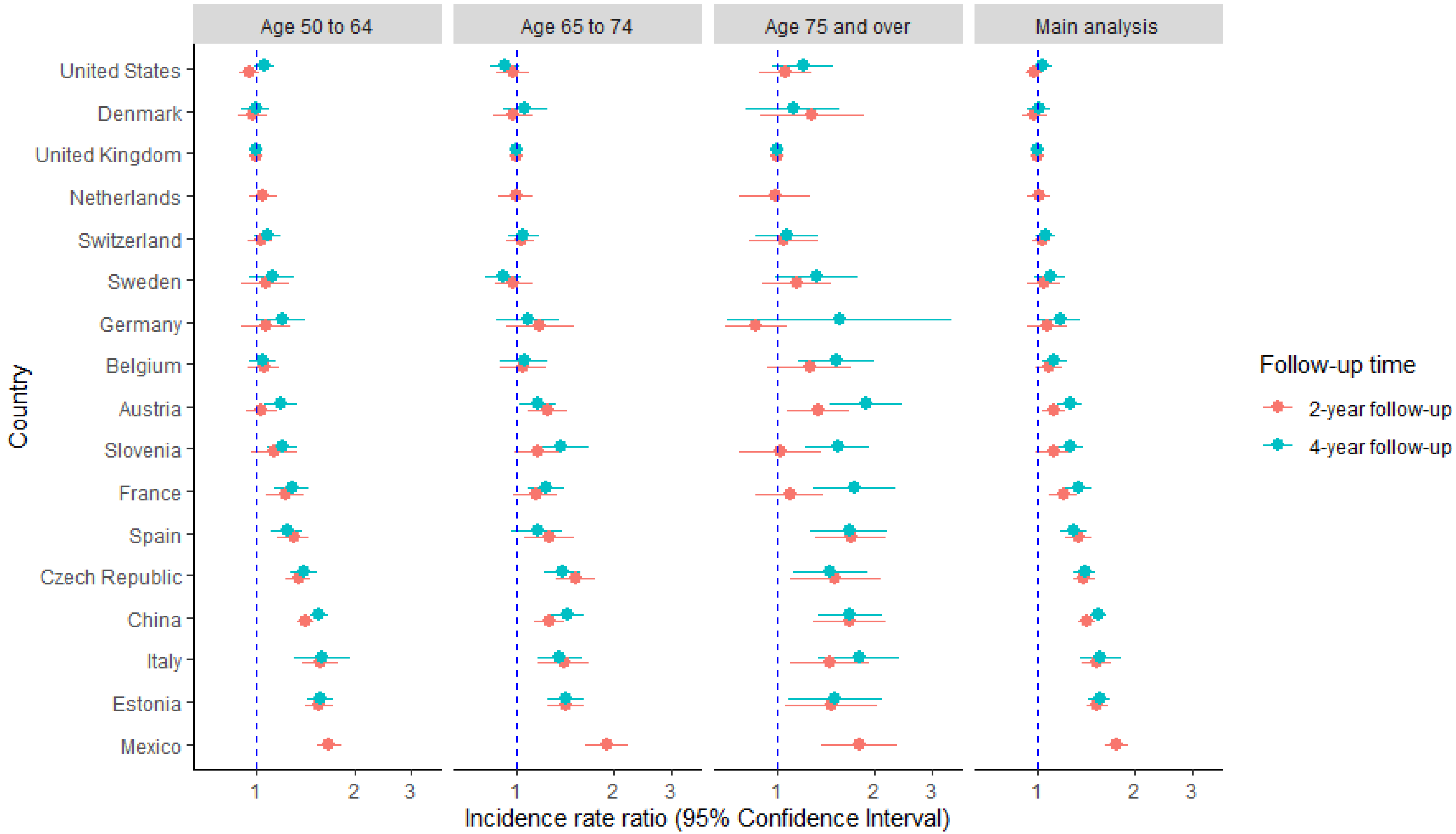

Figure 7 Age stratified analyses of accumulation of FI deficits: non-zero-state population

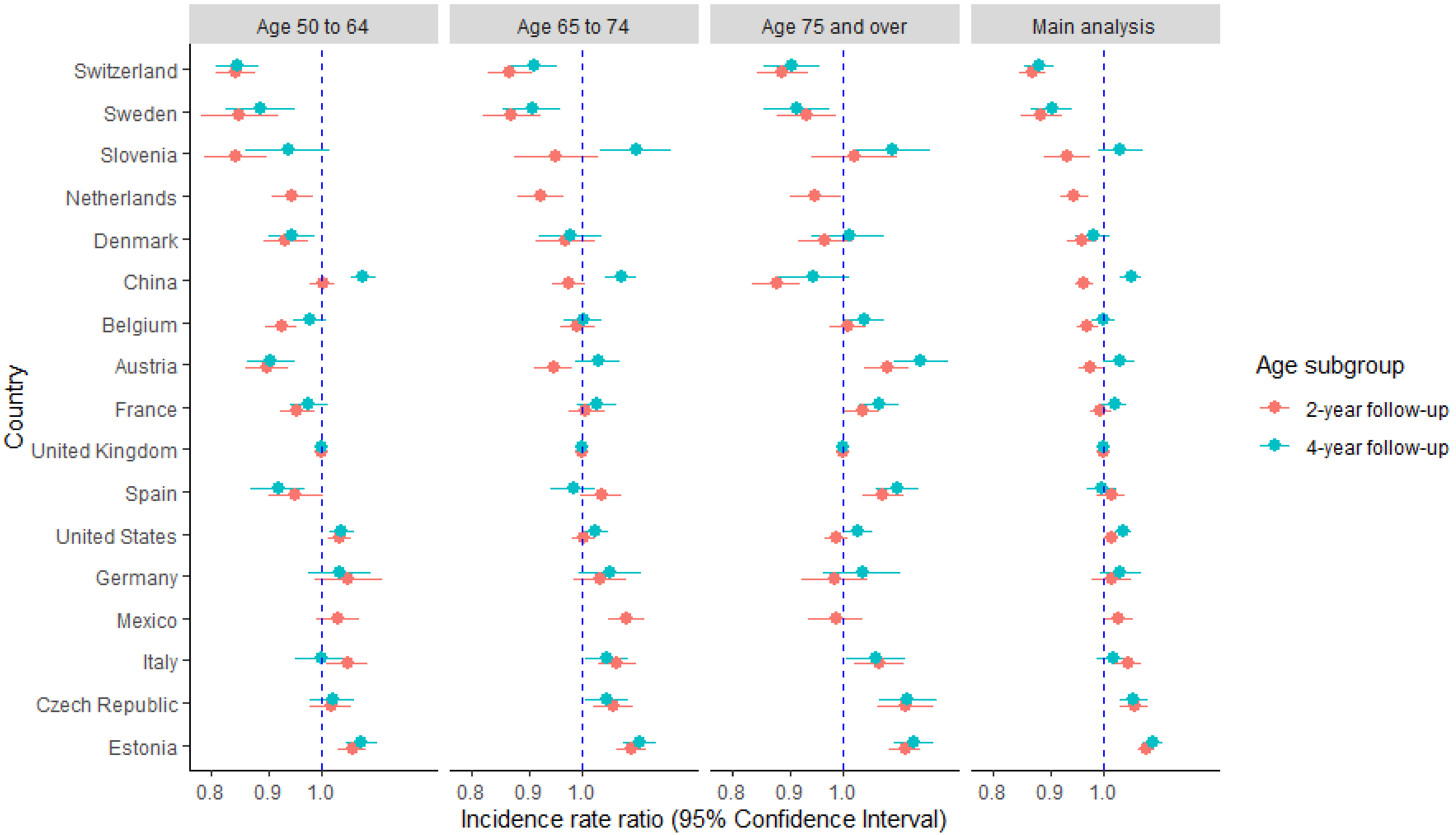

Sex stratified results

Figure 8 Mortality analysis by sex, 2-year follow-up

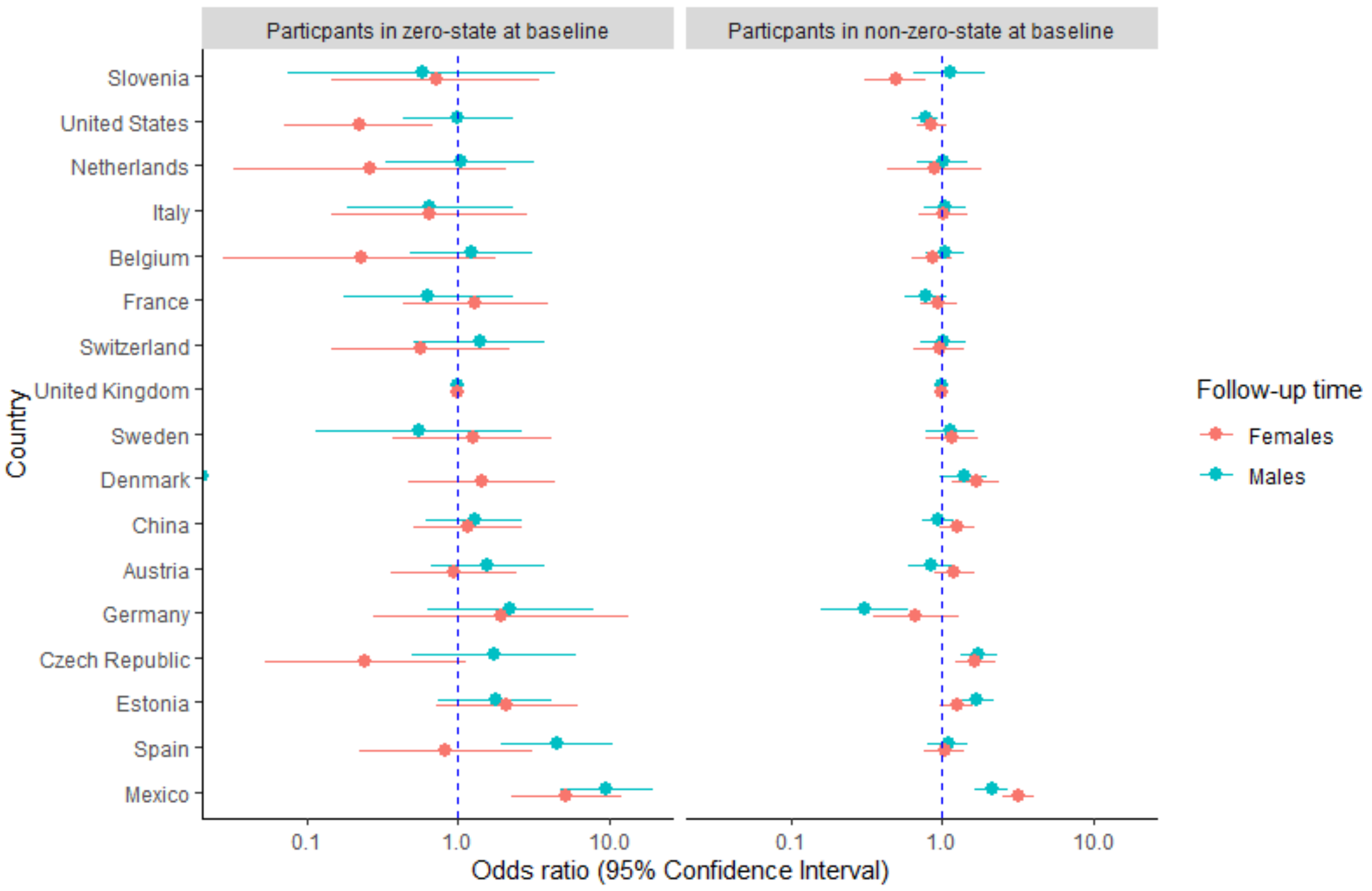

Figure 9 Mortality analysis by sex, 4-year follow-up

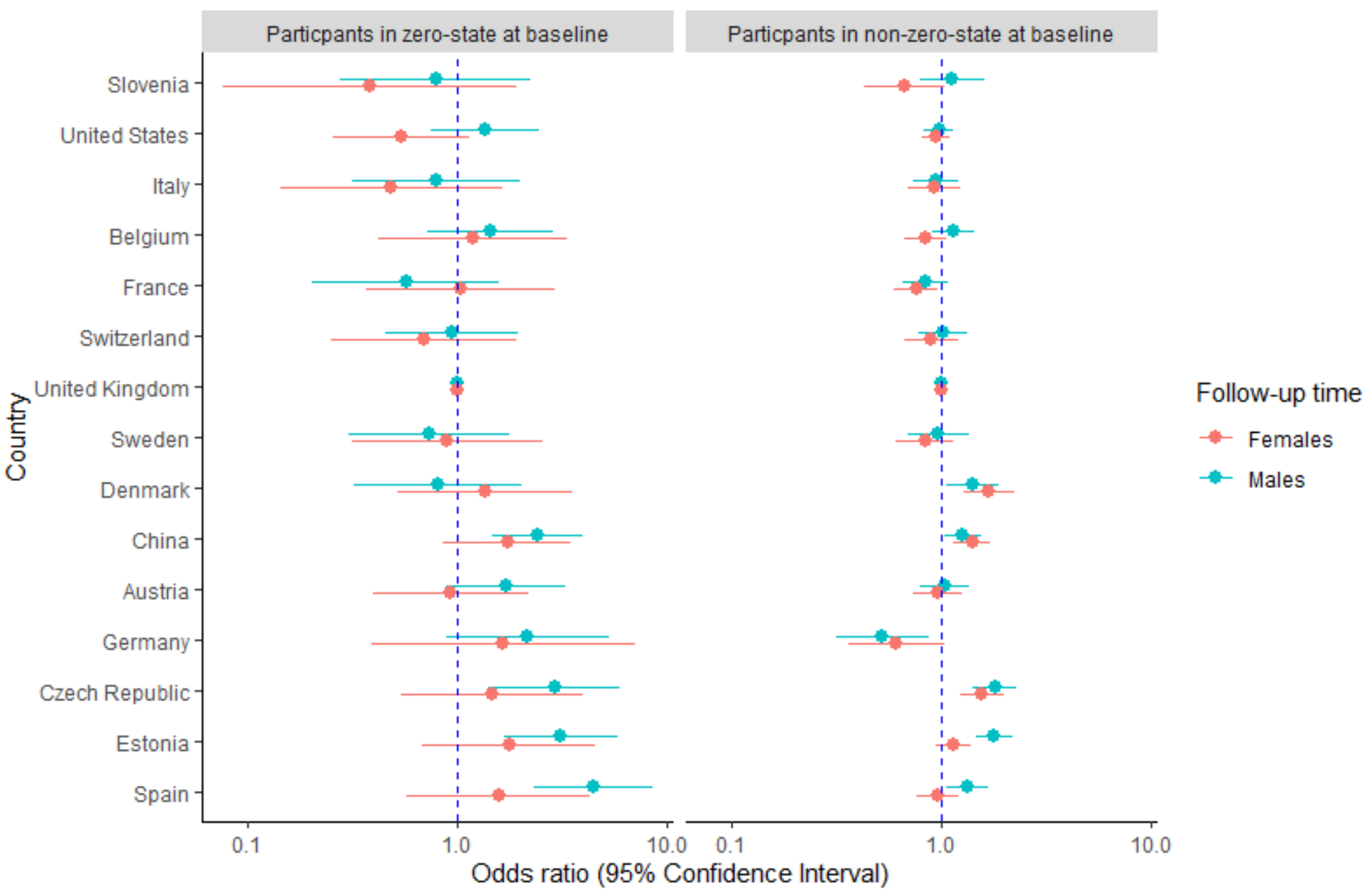

Figure 10 Accumulation of FI deficits by sex, 2-year follow-up

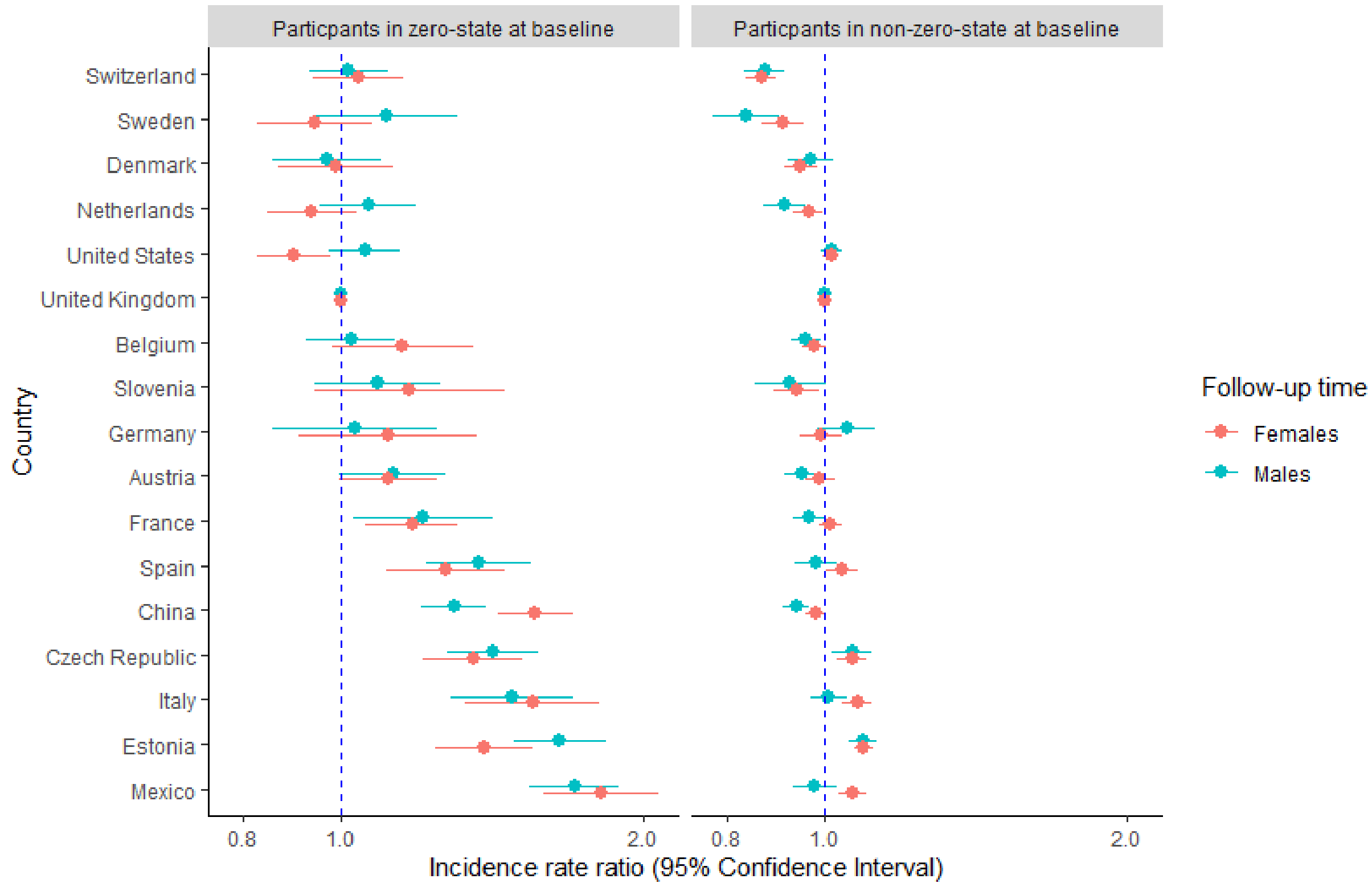

Figure 11 Accumulation of FI deficits by sex, 4-year follow-up

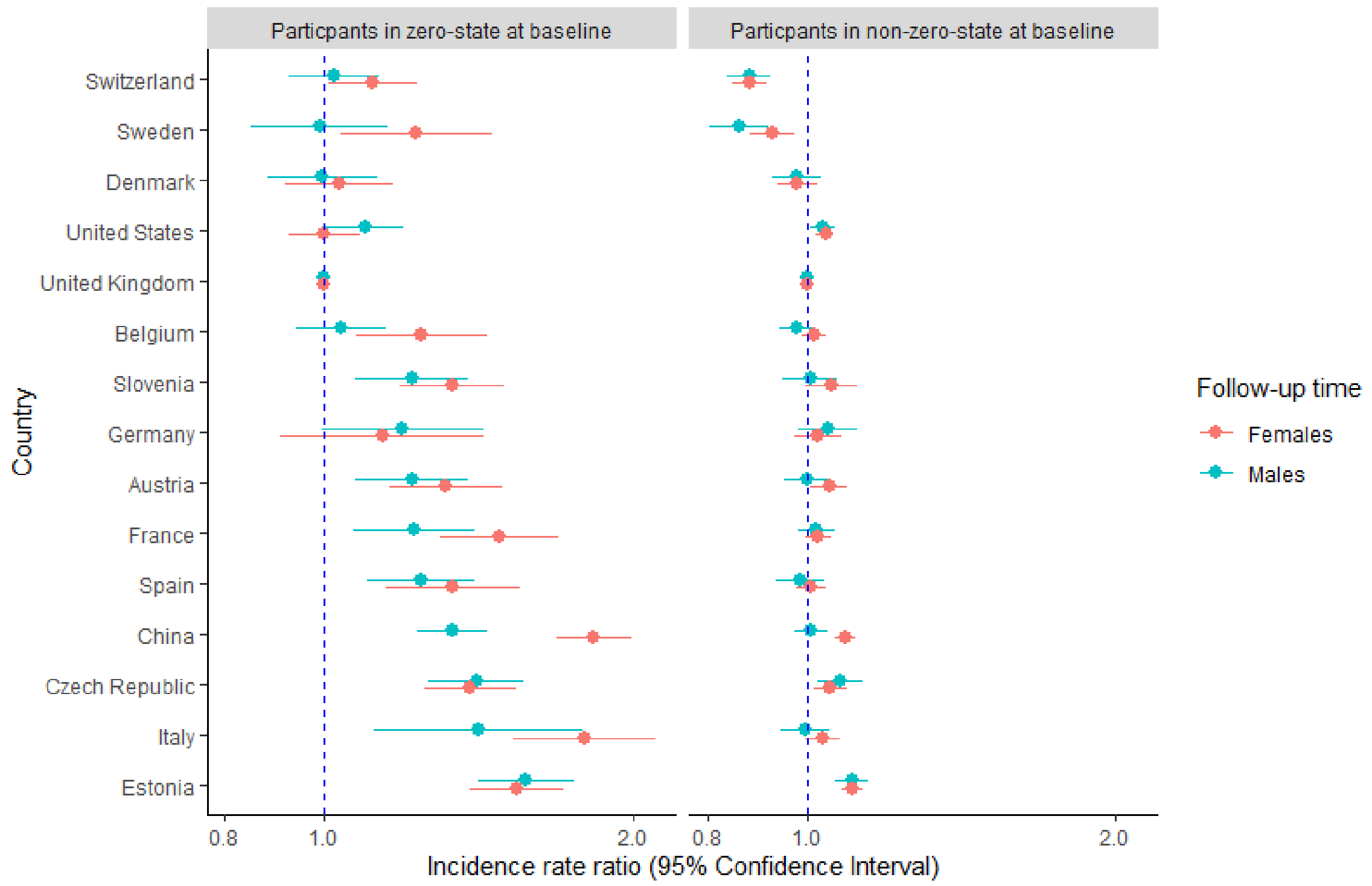

Adjustment for additional individual-level variables

Figure 12 Mortality analysis before and after additional adjustment, 2-year follow-up

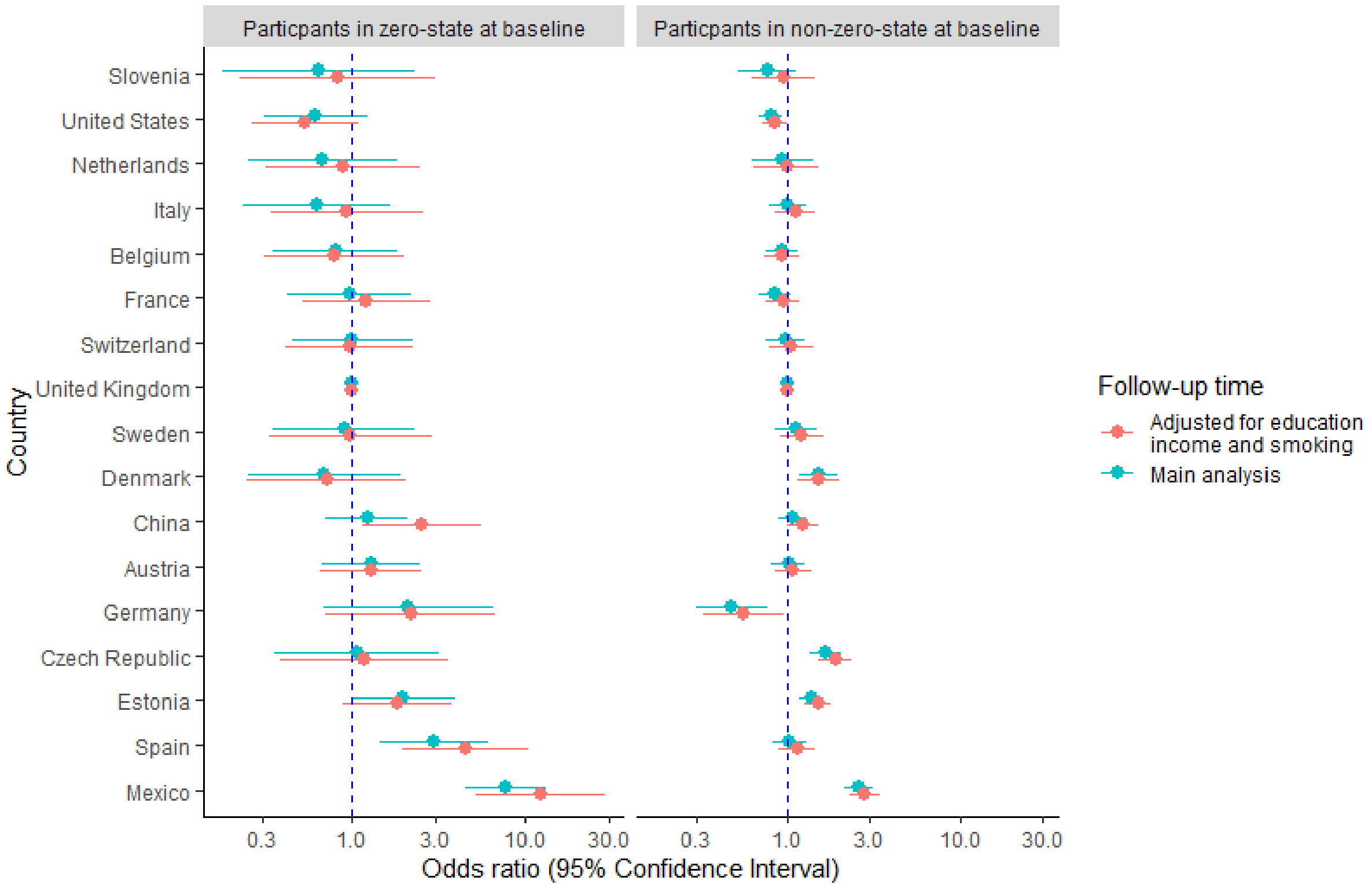

Figure 13 Mortality analysis before and after additional adjustment, 4-year follow-up

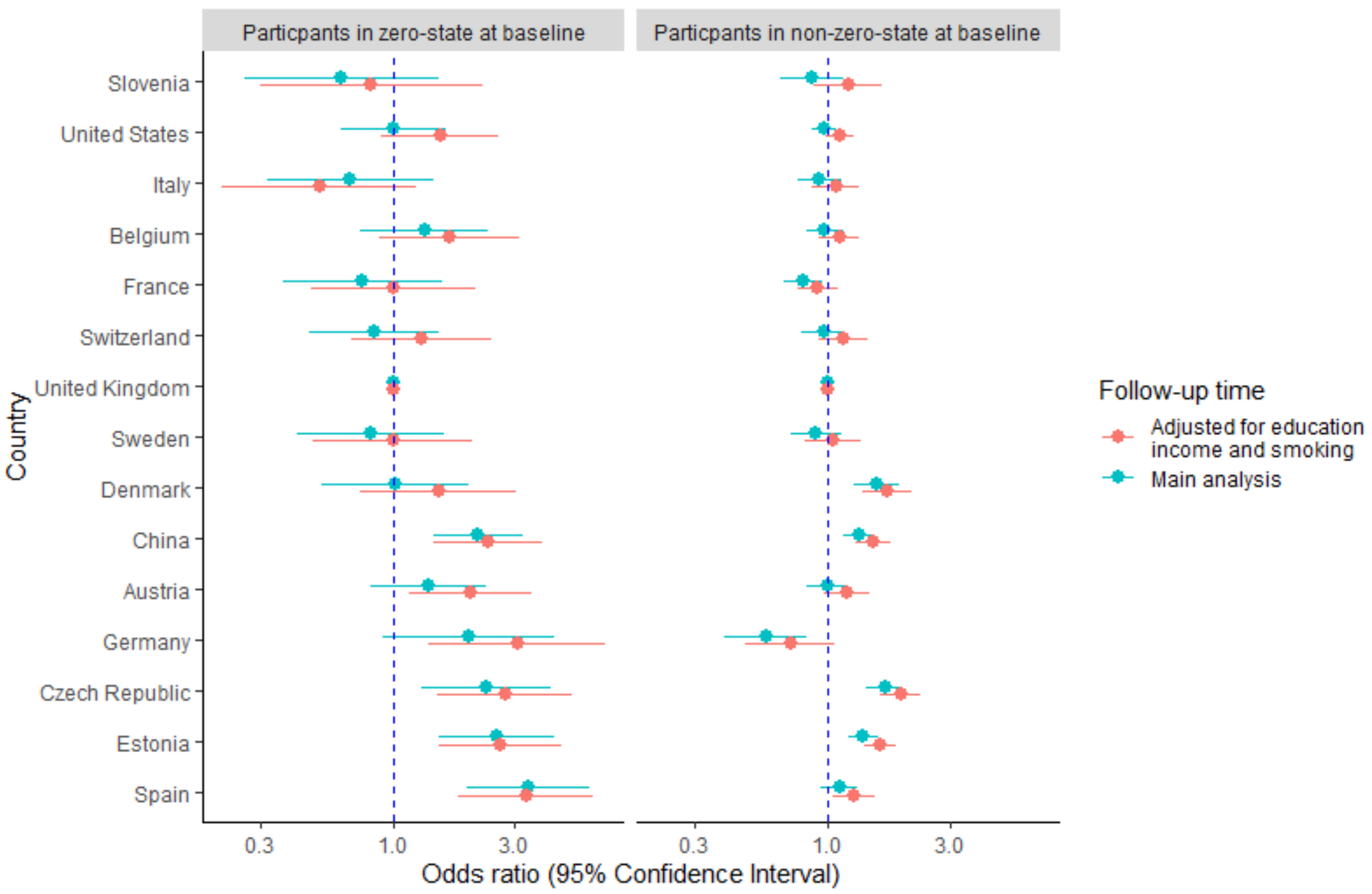

Figure 14 Accumulation of FI deficits before and after additional adjustment, 2-year follow-up

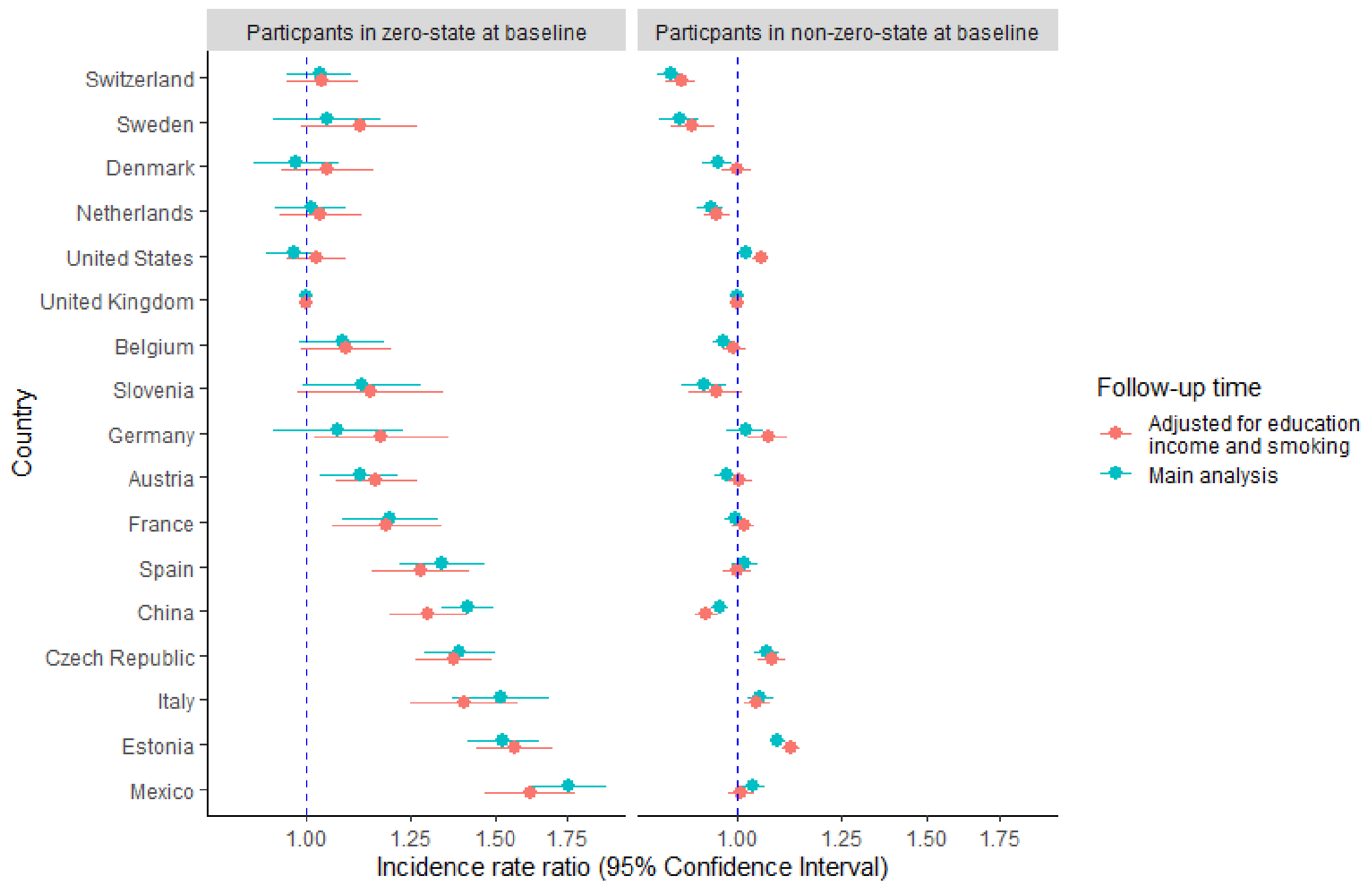

Figure 15 Accumulation of FI deficits before and after additional adjustment, 4-year follow-up

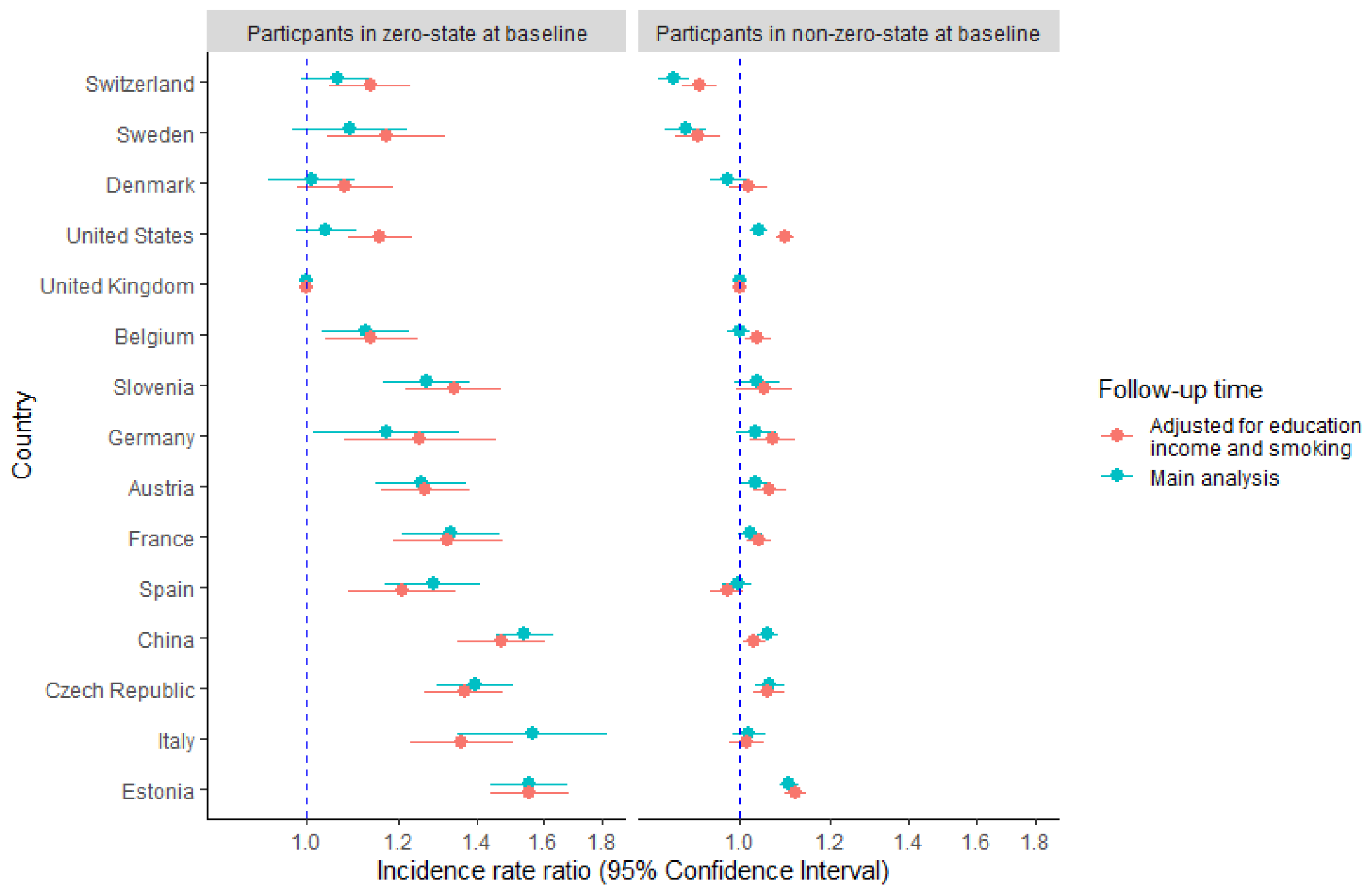

Deficit-level missing data

Table 6 Missing data: Difficulty raising arms above head

Country	Men age 50-59	Women age 50-59	Men age 60-69	Women age 60-69	Men age 70-79	Women age 70-79	Men age 80 and over	Women age 80 and over
United Kingdom	0%	0%	0%	0%	0%	0%	0%	0%
Austria	0%	0%	0%	0%	0%	0%	0%	0%
Belgium	0%	0%	0%	0%	0%	0%	0%	0%
China	0%	0%	0%	0%	0%	0.10%	0.40%	0.30%
Czech Republic	0%	0%	0%	0%	0%	0%	0%	0%
Denmark	0%	0%	0%	0%	0%	0%	0%	0%
Estonia	0%	0%	0%	0%	0%	0%	0%	0%
France	0%	0%	0%	0%	0%	0%	0%	0%
Germany	0%	0%	0%	0%	0%	0%	0%	0%
Italy	0%	0%	0%	0%	0%	0%	0%	0%
Mexico	0%	0%	0%	0%	0.10%	0%	0.20%	0%
Netherlands	0%	0%	0%	0%	0%	0%	0%	0%
Slovenia	0%	0%	0%	0%	0%	0%	0%	0%
Spain	0%	0%	0%	0%	0%	0%	0%	0%
Sweden	0%	0%	0%	0%	0%	0%	0%	0%
Switzerland	0%	0%	0%	0%	0%	0%	0%	0%
United States	0.10%	0%	0%	0%	0%	0.20%	0.20%	0.20%

Table 7 Missing data: Arthritis

Country	Men age 50-59	Women age 50-59	Men age 60-69	Women age 60-69	Men age 70-79	Women age 70-79	Men age 80 and over	Women age 80 and over
United Kingdom	0%	0%	0%	0.10%	0%	0%	0.20%	0%
Austria	0%	0%	0%	0%	0%	0%	0%	0%
Belgium	0%	0%	0%	0%	0%	0%	0%	0%
China	0.30%	0.20%	0.10%	0.20%	0.30%	0.20%	0%	0.70%
Czech Republic	0%	0%	0%	0%	0%	0%	0.50%	0%
Denmark	0%	0%	0%	0%	0%	0%	0%	0%
Estonia	0%	0%	0%	0%	0%	0%	0%	0%
France	0%	0%	0%	0%	0%	0%	0%	0%
Germany	0%	0%	0%	0%	0%	0%	0%	0%
Italy	0%	0%	0%	0%	0%	0%	0%	0%

Mexico	0.20%	0.20%	0.20%	0.10%	0.10%	0.10%	0%	0%
Netherlands	0%	0%	0%	0%	0%	0%	0%	0%
Slovenia	0%	0%	0%	0%	0%	0%	0%	0%
Spain	0%	0%	0%	0%	0%	0%	0%	0%
Sweden	0%	0%	0%	0%	0%	0%	0%	0%
Switzerland	0%	0%	0%	0%	0%	0%	0%	0%
United States	0.10%	0.10%	0.10%	0.20%	0.20%	0.10%	0.20%	0.30%

Table 8 Difficulty bathing

Country	Men age 50-59	Women age 50-59	Men age 60-69	Women age 60-69	Men age 70-79	Women age 70-79	Men age 80 and over	Women age 80 and over
United Kingdom	0%	0%	0%	0%	0%	0%	0%	0%
Austria	0%	0%	0%	0%	0%	0%	0%	0%
Belgium	0%	0%	0%	0%	0%	0%	0%	0%
China	0.70%	0.60%	0.50%	0.70%	0.80%	0.30%	0.40%	0.30%
Czech Republic	0%	0%	0%	0%	0%	0%	0%	0%
Denmark	0%	0%	0%	0%	0%	0%	0%	0%
Estonia	0%	0%	0%	0%	0%	0%	0%	0%
France	0%	0%	0%	0%	0%	0%	0%	0%
Germany	0%	0%	0%	0%	0%	0%	0%	0%
Italy	0%	0%	0%	0%	0%	0%	0%	0%
Mexico	0.10%	0.50%	0.50%	0.30%	0.20%	0.30%	0.60%	0.30%
Netherlands	0%	0%	0%	0%	0%	0%	0%	0%
Slovenia	0%	0%	0%	0%	0%	0%	0%	0%
Spain	0%	0%	0%	0%	0%	0%	0%	0%
Sweden	0%	0%	0%	0%	0%	0%	0%	0%
Switzerland	0%	0%	0%	0%	0%	0%	0%	0%
United States	0.10%	0%	0%	0%	0%	0%	0%	0.10%

Table 9 Missing data: Difficulty getting in/out of bed

Country	Men age 50-59	Women age 50-59	Men age 60-69	Women age 60-69	Men age 70-79	Women age 70-79	Men age 80 and over	Women age 80 and over
United Kingdom	0%	0%	0%	0%	0%	0%	0%	0%
Austria	0%	0%	0%	0%	0%	0%	0%	0%
Belgium	0%	0%	0%	0%	0%	0%	0%	0%
China	0.60%	0.60%	0.30%	0.60%	0.60%	0.30%	0%	0%

Czech Republic	0%	0%	0%	0%	0%	0%	0%	0%
Denmark	0%	0%	0%	0%	0%	0%	0%	0%
Estonia	0%	0%	0%	0%	0%	0%	0%	0%
France	0%	0%	0%	0%	0%	0%	0%	0%
Germany	0%	0%	0%	0%	0%	0%	0%	0%
Italy	0%	0%	0%	0%	0%	0%	0%	0%
Mexico	0%	0.30%	0.40%	0.20%	0.20%	0.30%	0.80%	0.20%
Netherlands	0%	0%	0%	0%	0%	0%	0%	0%
Slovenia	0%	0%	0%	0%	0%	0%	0%	0%
Spain	0%	0%	0%	0%	0%	0%	0%	0%
Sweden	0%	0%	0%	0%	0%	0%	0%	0%
Switzerland	0%	0%	0%	0%	0%	0%	0%	0%
United States	0%	0%	0%	0%	0%	0%	0.20%	0.10%

Table 10 Missing data: Hypertension

**Country**	**Men age 50-59**	**Women age 50-59**	**Men age 60-69**	**Women age 60-69**	**Men age 70-79**	**Women age 70-79**	**Men age 80 and over**	**Women age 80 and over**
United Kingdom	0%	0%	0%	0%	0%	0%	0%	0%
Austria	0%	0%	0%	0%	0%	0%	0%	0%
Belgium	0%	0%	0%	0%	0%	0%	0%	0%
China	0.50%	0.40%	0.30%	0.30%	0.30%	0.60%	0%	0.70%
Czech Republic	0%	0%	0%	0%	0%	0%	0%	0%
Denmark	0%	0%	0%	0%	0%	0%	0%	0%
Estonia	0%	0%	0%	0%	0%	0%	0%	0%
France	0%	0%	0%	0%	0%	0%	0%	0%
Germany	0%	0%	0%	0%	0%	0%	0%	0%
Italy	0%	0%	0%	0%	0%	0%	0%	0%
Mexico	0.30%	0.20%	0%	0.20%	0.20%	0.10%	0%	0.30%
Netherlands	0%	0%	0%	0%	0%	0%	0%	0%
Slovenia	0%	0%	0%	0%	0%	0%	0%	0%
Spain	0%	0%	0%	0%	0%	0%	0%	0%
Sweden	0%	0%	0%	0%	0%	0%	0%	0%
Switzerland	0%	0%	0%	0%	0%	0%	0%	0%
United States	0.10%	0%	0.20%	0.20%	0.20%	0.10%	0.70%	0.40%

Table 11 Missing data: Cancer

Country	Men age 50-59	Women age 50-59	Men age 60-69	Women age 60-69	Men age 70-79	Women age 70-79	Men age 80 and over	Women age 80 and over
United Kingdom	0%	0%	0%	0.10%	0%	0%	0%	0%
Austria	0%	0%	0%	0%	0%	0%	0%	0%
Belgium	0%	0%	0%	0%	0%	0%	0%	0%
China	0.60%	0.30%	0.40%	0.40%	0.40%	0.40%	0%	0.30%
Czech Republic	0%	0%	0%	0%	0%	0%	0.50%	0%
Denmark	0%	0%	0%	0%	0%	0%	0%	0%
Estonia	0%	0%	0%	0%	0%	0%	0%	0%
France	0%	0%	0%	0%	0%	0%	0%	0%
Germany	0%	0%	0%	0%	0%	0%	0%	0%
Italy	0%	0%	0%	0%	0%	0%	0%	0%
Mexico	0.20%	0.20%	0.20%	0%	0.10%	0.10%	0.20%	0.30%
Netherlands	0%	0%	0%	0%	0%	0%	0%	0%
Slovenia	0%	0%	0%	0%	0%	0%	0%	0%
Spain	0%	0%	0%	0%	0%	0%	0%	0%
Sweden	0%	0%	0%	0%	0%	0%	0%	0%
Switzerland	0%	0%	0%	0%	0%	0%	0%	0%
United States	0%	0.10%	0.10%	0.30%	0.50%	0.30%	0.10%	0.30%

Table 12 Missing data: Difficulty carrying 10 pounds or more

Country	Men age 50-59	Women age 50-59	Men age 60-69	Women age 60-69	Men age 70-79	Women age 70-79	Men age 80 and over	Women age 80 and over
United Kingdom	0%	0%	0%	0%	0%	0%	0%	0%
Austria	0%	0%	0%	0%	0%	0%	0%	0%
Belgium	0%	0%	0%	0%	0%	0%	0%	0%
China	0%	0%	0%	0.30%	0%	0%	0%	0%
Czech Republic	0%	0%	0%	0%	0%	0%	0%	0%
Denmark	0%	0%	0%	0%	0%	0%	0%	0%
Estonia	0%	0%	0%	0%	0%	0%	0%	0%
France	0%	0%	0%	0%	0%	0%	0%	0%
Germany	0%	0%	0%	0%	0%	0%	0%	0%
Italy	0%	0%	0%	0%	0%	0%	0%	0%
Mexico	0.40%	1%	0.30%	2%	0.80%	2.70%	4.50%	6.90%
Netherlands	0%	0%	0%	0%	0%	0%	0%	0%

Slovenia	0%	0%	0%	0%	0%	0%	0%	0%
Spain	0%	0%	0%	0%	0%	0%	0%	0%
Sweden	0%	0%	0%	0%	0%	0%	0%	0%
Switzerland	0%	0%	0%	0%	0%	0%	0%	0%
United States	0%	0%	0%	0.10%	0.10%	0.10%	0.20%	0.30%

Table 13 Missing data: Difficulty rising from a chair

Country	Men age 50-59	Women age 50-59	Men age 60-69	Women age 60-69	Men age 70-79	Women age 70-79	Men age 80 and over	Women age 80 and over
United Kingdom	0%	0%	0%	0%	0%	0%	0%	0%
Austria	0%	0%	0%	0%	0%	0%	0%	0%
Belgium	0%	0%	0%	0%	0%	0%	0%	0%
China	0.10%	0%	0%	0%	0%	0.10%	0%	0.30%
Czech Republic	0%	0%	0%	0%	0%	0%	0%	0%
Denmark	0%	0%	0%	0%	0%	0%	0%	0%
Estonia	0%	0%	0%	0%	0%	0%	0%	0%
France	0%	0%	0%	0%	0%	0%	0%	0%
Germany	0%	0%	0%	0%	0%	0%	0%	0%
Italy	0%	0%	0%	0%	0%	0%	0%	0%
Mexico	0.10%	0.20%	0.20%	0.10%	0%	0.10%	0.40%	0%
Netherlands	0%	0%	0%	0%	0%	0%	0%	0%
Slovenia	0%	0%	0%	0%	0%	0%	0%	0%
Spain	0%	0%	0%	0%	0%	0%	0%	0%
Sweden	0%	0%	0%	0%	0%	0%	0%	0%
Switzerland	0%	0%	0%	0%	0%	0%	0%	0%
United States	0.10%	0.10%	0.10%	0.10%	0.20%	0.20%	0.10%	0.10%

Table 14 Missing data: Difficulty picking up a coin

Country	Men age 50-59	Women age 50-59	Men age 60-69	Women age 60-69	Men age 70-79	Women age 70-79	Men age 80 and over	Women age 80 and over
United Kingdom	0%	0%	0%	0%	0%	0%	0%	0%
Austria	0%	0%	0%	0%	0%	0%	0%	0%
Belgium	0%	0%	0%	0%	0%	0%	0%	0%
China	0.10%	0.10%	0%	0.10%	0%	0%	0.40%	0%
Czech Republic	0%	0%	0%	0%	0%	0%	0%	0%

Denmark	0%	0%	0%	0%	0%	0%	0%	0%
Estonia	0%	0%	0%	0%	0%	0%	0%	0%
France	0%	0%	0%	0%	0%	0%	0%	0%
Germany	0%	0%	0%	0%	0%	0%	0%	0%
Italy	0%	0%	0%	0%	0%	0%	0%	0%
Mexico	0%	0%	0%	0%	0.20%	0.10%	0.20%	0.50%
Netherlands	0%	0%	0%	0%	0%	0%	0%	0%
Slovenia	0%	0%	0%	0%	0%	0%	0%	0%
Spain	0%	0%	0%	0%	0%	0%	0%	0%
Sweden	0%	0%	0%	0%	0%	0%	0%	0%
Switzerland	0%	0%	0%	0%	0%	0%	0%	0%
United States	0%	0.10%	0%	0.10%	0.10%	0.10%	0.30%	0.70%

Table 15 Missing data: Depression

**Country**	**Men age 50-59**	**Women age 50-59**	**Men age 60-69**	**Women age 60-69**	**Men age 70-79**	**Women age 70-79**	**Men age 80 and over**	**Women age 80 and over**
United Kingdom	4.60%	2.80%	4.90%	2.80%	4%	2.80%	9.20%	12.20%
Austria	0.90%	0.50%	1.10%	0.50%	0.60%	0.70%	1.60%	1.60%
Belgium	0.20%	0.60%	0.10%	0%	0.20%	0.20%	2%	1.60%
China	12.20%	6.10%	7.20%	7%	7.30%	8.40%	19.40%	25.60%
Czech Republic	1.60%	0.70%	1.30%	0.60%	2.20%	0.60%	1.10%	3.20%
Denmark	0.30%	0%	0.30%	0%	0.50%	1.40%	2%	3.20%
Estonia	1.50%	0.60%	1.20%	0.50%	2.20%	0.60%	0.80%	2.70%
France	0.80%	0.50%	0.60%	0.40%	0.90%	0.60%	4%	2.10%
Germany	0.90%	0.50%	0.30%	0%	1.10%	0.40%	2.40%	2%
Italy	1.10%	1.40%	1%	1%	1.70%	1.60%	3%	3.20%
Mexico	0.20%	0.10%	0.20%	0.20%	0%	0.10%	0.20%	0.20%
Netherlands	0%	0%	0.60%	0.30%	0.40%	0.30%	0.90%	0.60%
Slovenia	0.50%	0.20%	0.50%	0%	0.40%	0.30%	0%	1.10%
Spain	0.20%	0.20%	0.60%	0.70%	2.30%	1%	4.10%	4.90%
Sweden	0%	0%	0.30%	0.20%	0.40%	0.30%	1.40%	4.40%
Switzerland	0.60%	0.30%	0.20%	0%	0%	0.50%	0.70%	1.30%
United States	4.20%	1.50%	7.20%	2.30%	9.80%	4.80%	15.50%	20%

Table 16 Missing data: Diabetes

**Country**	**Men age 50-**	**Women age 50-**	**Men age 60-**	**Women age 60-**	**Men age 70-**	**Women age 70-**	**Men age 80**	**Women age 80**

	59	59	69	69	79	79	and over	and over
United Kingdom	0%	0%	0%	0%	0%	0%	0%	0%
Austria	0%	0%	0%	0%	0%	0%	0%	0%
Belgium	0%	0%	0%	0%	0%	0%	0%	0%
China	0.80%	1%	0.80%	0.80%	0.40%	0.80%	0%	1.40%
Czech Republic	0%	0%	0%	0%	0%	0%	0.50%	0%
Denmark	0%	0%	0%	0%	0%	0%	0%	0%
Estonia	0%	0%	0%	0%	0%	0%	0%	0%
France	0%	0%	0%	0%	0%	0%	0%	0%
Germany	0%	0%	0%	0%	0%	0%	0%	0%
Italy	0%	0%	0%	0%	0%	0%	0%	0%
Mexico	0.30%	0.20%	0%	0.10%	0.10%	0.20%	0%	0.30%
Netherlands	0%	0%	0%	0%	0%	0%	0%	0%
Slovenia	0%	0%	0%	0%	0%	0%	0%	0%
Spain	0%	0%	0%	0%	0%	0%	0%	0%
Sweden	0%	0%	0%	0%	0%	0%	0%	0%
Switzerland	0%	0%	0%	0%	0%	0%	0%	0%
United States	0%	0.10%	0%	0.10%	0.20%	0.30%	0.20%	0.30%

Table 17 Missing data: Difficulty dressing

Country	Men age 50-59	Women age 50-59	Men age 60-69	Women age 60-69	Men age 70-79	Women age 70-79	Men age 80 and over	Women age 80 and over
United Kingdom	0%	0%	0%	0%	0%	0%	0%	0%
Austria	0%	0%	0%	0%	0%	0%	0%	0%
Belgium	0%	0%	0%	0%	0%	0%	0%	0%
China	0.60%	0.60%	0.30%	0.60%	0.60%	0.20%	0%	0%
Czech Republic	0%	0%	0%	0%	0%	0%	0%	0%
Denmark	0%	0%	0%	0%	0%	0%	0%	0%
Estonia	0%	0%	0%	0%	0%	0%	0%	0%
France	0%	0%	0%	0%	0%	0%	0%	0%
Germany	0%	0%	0%	0%	0%	0%	0%	0%
Italy	0%	0%	0%	0%	0%	0%	0%	0%
Mexico	0%	0.10%	0%	0.10%	0.10%	0.20%	0%	0.30%
Netherlands	0%	0%	0%	0%	0%	0%	0%	0%
Slovenia	0%	0%	0%	0%	0%	0%	0%	0%
Spain	0%	0%	0%	0%	0%	0%	0%	0%
Sweden	0%	0%	0%	0%	0%	0%	0%	0%
Switzerland	0%	0%	0%	0%	0%	0%	0%	0%

United States	0%	0%	0%	0%	0%	0%	0%	0.10%

Table 18 Missing data: Difficulty eating

Country	Men age 50-59	Women age 50-59	Men age 60-69	Women age 60-69	Men age 70-79	Women age 70-79	Men age 80 and over	Women age 80 and over
United Kingdom	0%	0%	0%	0%	0%	0%	0%	0%
Austria	0%	0%	0%	0%	0%	0%	0%	0%
Belgium	0%	0%	0%	0%	0%	0%	0%	0%
China	0.60%	0.60%	0.30%	0.60%	0.60%	0.20%	0%	0%
Czech Republic	0%	0%	0%	0%	0%	0%	0%	0%
Denmark	0%	0%	0%	0%	0%	0%	0%	0%
Estonia	0%	0%	0%	0%	0%	0%	0%	0%
France	0%	0%	0%	0%	0%	0%	0%	0%
Germany	0%	0%	0%	0%	0%	0%	0%	0%
Italy	0%	0%	0%	0%	0%	0%	0%	0%
Mexico	0.20%	0.20%	0.60%	0.20%	0.70%	0.30%	0.40%	0.20%
Netherlands	0%	0%	0%	0%	0%	0%	0%	0%
Slovenia	0%	0%	0%	0%	0%	0%	0%	0%
Spain	0%	0%	0%	0%	0%	0%	0%	0%
Sweden	0%	0%	0%	0%	0%	0%	0%	0%
Switzerland	0%	0%	0%	0%	0%	0%	0%	0%
United States	0%	0%	0%	0%	0%	0%	0.10%	0.20%

Table 19 Missing data: Fallen in the past 2 years

Country	Men age 50-59	Women age 50-59	Men age 60-69	Women age 60-69	Men age 70-79	Women age 70-79	Men age 80 and over	Women age 80 and over
United Kingdom	0%	0%	0%	0%	0%	0%	0%	0%
Austria	0%	0%	0.30%	0.20%	0.20%	0.30%	0%	0.30%
Belgium	0%	0.10%	0%	0%	0.20%	0%	0%	0%
China	10.70%	4.60%	5.80%	4.50%	5.40%	5.30%	13.80%	20.10%
Czech Republic	0.50%	0.30%	0%	0.20%	0.60%	0.30%	0%	0%
Denmark	0%	0%	0%	0%	0%	0%	0%	0%
Estonia	0.10%	0%	0%	0%	0%	0%	0%	0%
France	0%	0%	0%	0%	0%	0%	0%	0%

Germany	0.90%	0.50%	0%	0%	0%	0%	0%	0%
Italy	0.80%	1.20%	0.70%	0.70%	0.60%	0.60%	0%	0%
Mexico	0%	0%	0%	0%	0%	0%	0%	0%
Netherlands	0%	0%	0%	0%	0%	0%	0%	0%
Slovenia	0%	0%	0%	0%	0.40%	0%	0%	0%
Spain	0%	0.20%	0%	0%	0%	0%	0%	0%
Sweden	0%	0%	0%	0%	0%	0%	0%	0%
Switzerland	0.20%	0.20%	0%	0%	0%	0%	0%	0%
United States	100%	100%	55.20%	55%	0%	0.40%	0.40%	0.30%

Table 20 Missing data: Hearing difficulty

Country	Men age 50-59	Women age 50-59	Men age 60-69	Women age 60-69	Men age 70-79	Women age 70-79	Men age 80 and over	Women age 80 and over
United Kingdom	0%	0%	0%	0%	0%	0%	0%	0%
Austria	0%	0%	0.30%	0.20%	0.20%	0.30%	0%	0.30%
Belgium	0%	0.10%	0%	0%	0.20%	0%	0%	0%
China	0%	0.10%	0.10%	0%	0%	0.10%	0%	0%
Czech Republic	0.50%	0.30%	0%	0.20%	0.60%	0.30%	0%	0%
Denmark	0%	0%	0%	0%	0%	0%	0%	0%
Estonia	0.10%	0%	0%	0%	0%	0%	0%	0%
France	0%	0%	0%	0%	0%	0%	0%	0%
Germany	0.90%	0.50%	0%	0%	0%	0%	0%	0%
Italy	0.80%	1.20%	0.70%	0.70%	0.60%	0.60%	0%	0%
Mexico	0%	0%	0%	0%	0%	0%	0%	0%
Netherlands	0%	0%	0%	0%	0%	0%	0%	0%
Slovenia	0%	0%	0%	0%	0.40%	0%	0%	0%
Spain	0%	0.20%	0%	0%	0%	0%	0%	0%
Sweden	0%	0%	0%	0%	0%	0%	0%	0%
Switzerland	0.20%	0.20%	0%	0%	0%	0%	0%	0%
United States	0%	0%	0%	0.10%	0.10%	0.10%	0.30%	0%

Table 21 Missing data: Heart problems

Country	Men age 50-59	Women age 50-59	Men age 60-69	Women age 60-69	Men age 70-79	Women age 70-79	Men age 80 and over	Women age 80 and over

United Kingdom	0%	0%	0%	0%	0%	0%	0%	0%
Austria	0%	0%	0%	0%	0%	0%	0%	0%
Belgium	0%	0%	0%	0%	0%	0%	0%	0%
China	0.50%	0.40%	0.50%	0.60%	0.50%	0.40%	0%	0.70%
Czech Republic	0%	0%	0%	0%	0%	0%	0%	0%
Denmark	0%	0%	0%	0%	0%	0%	0%	0%
Estonia	0%	0%	0%	0%	0%	0%	0%	0%
France	0%	0%	0%	0%	0%	0%	0%	0%
Germany	0%	0%	0%	0%	0%	0%	0%	0%
Italy	0%	0%	0%	0%	0%	0%	0%	0%
Mexico	0.30%	0.10%	0.10%	0%	0.20%	0.10%	0.20%	0.20%
Netherlands	0%	0%	0%	0%	0%	0%	0%	0%
Slovenia	0%	0%	0%	0%	0%	0%	0%	0%
Spain	0%	0%	0%	0%	0%	0%	0%	0%
Sweden	0%	0%	0%	0%	0%	0%	0%	0%
Switzerland	0%	0%	0%	0%	0%	0%	0%	0%
United States	0%	0%	0.10%	0.20%	0.10%	0.30%	0.10%	0.20%

Table 22 Missing data: Lung problems

**Country**	**Men age 50-59**	**Women age 50-59**	**Men age 60-69**	**Women age 60-69**	**Men age 70-79**	**Women age 70-79**	**Men age 80 and over**	**Women age 80 and over**
United Kingdom	0%	0%	0%	0.10%	0%	0%	0%	0%
Austria	0%	0%	0%	0%	0%	0%	0%	0%
Belgium	0%	0%	0%	0%	0%	0%	0%	0%
China	0.40%	0.30%	0.30%	0.30%	0.10%	0.50%	0%	1%
Czech Republic	0%	0%	0%	0%	0%	0%	0.50%	0%
Denmark	0%	0%	0%	0%	0%	0%	0%	0%
Estonia	0%	0%	0%	0%	0%	0%	0%	0%
France	0%	0%	0%	0%	0%	0%	0%	0%
Germany	0%	0%	0%	0%	0%	0%	0%	0%
Italy	0%	0%	0%	0%	0%	0%	0%	0%
Mexico	0.20%	0.20%	0.20%	0%	0.20%	0.10%	0.20%	0.30%
Netherlands	0%	0%	0%	0%	0%	0%	0%	0%
Slovenia	0%	0%	0%	0%	0%	0%	0%	0%
Spain	0%	0%	0%	0%	0%	0%	0%	0%
Sweden	0%	0%	0%	0%	0%	0%	0%	0%
Switzerland	0%	0%	0%	0%	0%	0%	0%	0%

United States	0%	0%	0.10%	0.10%	0.20%	0.10%	0.30%	0.20%

Table 23 Missing data: Difficulty preparing meals

**Country**	**Men age 50-59**	**Women age 50-59**	**Men age 60-69**	**Women age 60-69**	**Men age 70-79**	**Women age 70-79**	**Men age 80 and over**	**Women age 80 and over**
United Kingdom	0%	0%	0%	0%	0%	0%	0%	0%
Austria	0%	0%	0%	0%	0%	0%	0%	0%
Belgium	0%	0%	0%	0%	0%	0%	0%	0%
China	0%	0%	0.10%	0%	0.10%	0%	0.40%	0.30%
Czech Republic	0%	0%	0%	0%	0%	0%	0%	0%
Denmark	0%	0%	0%	0%	0%	0%	0%	0%
Estonia	0%	0%	0%	0%	0%	0%	0%	0%
France	0%	0%	0%	0%	0%	0%	0%	0%
Germany	0%	0%	0%	0%	0%	0%	0%	0%
Italy	0%	0%	0%	0%	0%	0%	0%	0%
Mexico	7.20%	0.10%	9.30%	0.10%	12.10%	0.80%	19.60%	3.30%
Netherlands	0%	0%	0%	0%	0%	0%	0%	0%
Slovenia	0%	0%	0%	0%	0%	0%	0%	0%
Spain	0%	0%	0%	0%	0%	0%	0%	0%
Sweden	0%	0%	0%	0%	0%	0%	0%	0%
Switzerland	0%	0%	0%	0%	0%	0%	0%	0%
United States	0.10%	0%	0%	0%	0.10%	0%	0.10%	0.10%

Table 24 Missing data: Difficulty managing medication

**Country**	**Men age 50-59**	**Women age 50-59**	**Men age 60-69**	**Women age 60-69**	**Men age 70-79**	**Women age 70-79**	**Men age 80 and over**	**Women age 80 and over**
United Kingdom	0%	0%	0%	0%	0%	0%	0%	0%
Austria	0%	0%	0%	0%	0%	0%	0%	0%
Belgium	0%	0%	0%	0%	0%	0%	0%	0%
China	0.10%	0%	0%	0.20%	0.20%	0.20%	0%	1%
Czech Republic	0%	0%	0%	0%	0%	0%	0%	0%
Denmark	0%	0%	0%	0%	0%	0%	0%	0%
Estonia	0%	0%	0%	0%	0%	0%	0%	0%
France	0%	0%	0%	0%	0%	0%	0%	0%

Germany	0%	0%	0%	0%	0%	0%	0%	0%
Italy	0%	0%	0%	0%	0%	0%	0%	0%
Mexico	1.60%	0.70%	1.80%	0.60%	1.60%	0.60%	2%	1.60%
Netherlands	0%	0%	0%	0%	0%	0%	0%	0%
Slovenia	0%	0%	0%	0%	0%	0%	0%	0%
Spain	0%	0%	0%	0%	0%	0%	0%	0%
Sweden	0%	0%	0%	0%	0%	0%	0%	0%
Switzerland	0%	0%	0%	0%	0%	0%	0%	0%
United States	1.90%	1%	1.30%	0.90%	1.40%	1%	1.40%	1.20%

Table 25 Missing data: Difficulty managing money

Country	Men age 50-59	Women age 50-59	Men age 60-69	Women age 60-69	Men age 70-79	Women age 70-79	Men age 80 and over	Women age 80 and over
United Kingdom	0%	0%	0%	0%	0%	0%	0%	0%
Austria	0%	0%	0%	0%	0%	0%	0%	0%
Belgium	0%	0%	0%	0%	0%	0%	0%	0%
China	0.10%	0.30%	0.10%	0.30%	0.50%	0.60%	0.40%	0.30%
Czech Republic	0%	0%	0%	0%	0%	0%	0%	0%
Denmark	0%	0%	0%	0%	0%	0%	0%	0%
Estonia	0%	0%	0%	0%	0%	0%	0%	0%
France	0%	0%	0%	0%	0%	0%	0%	0%
Germany	0%	0%	0%	0%	0%	0%	0%	0%
Italy	0%	0%	0%	0%	0%	0%	0%	0%
Mexico	0.10%	0.20%	0.20%	0.40%	0.20%	0.60%	1%	1.10%
Netherlands	0%	0%	0%	0%	0%	0%	0%	0%
Slovenia	0%	0%	0%	0%	0%	0%	0%	0%
Spain	0%	0%	0%	0%	0%	0%	0%	0%
Sweden	0%	0%	0%	0%	0%	0%	0%	0%
Switzerland	0%	0%	0%	0%	0%	0%	0%	0%
United States	0.10%	0.10%	0%	0%	0.10%	0%	0.10%	0.30%

Table 26 Missing data: Feels sad

Country	Men age 50-59	Women age 50-59	Men age 60-69	Women age 60-69	Men age 70-79	Women age 70-79	Men age 80 and over	Women age 80 and over
United Kingdom	4.60%	2.90%	4.90%	2.80%	3.90%	2.70%	9.50%	12.30%

Austria	2.50%	1.60%	2.40%	1.10%	1.70%	1%	2.10%	1.60%
Belgium	1%	0.40%	0.70%	0.20%	0.60%	0.50%	4.40%	4.70%
China	12.20%	5.30%	7.10%	6.10%	6.90%	7.40%	18.50%	23.10%
Czech Republic	2.20%	1%	2.80%	1.40%	3%	1.60%	2.70%	4.30%
Denmark	1.40%	0.20%	0.60%	0.30%	2.60%	1%	5.10%	4.40%
Estonia	3.90%	1.20%	3.50%	0.90%	3.80%	0.50%	2.50%	2.90%
France	2.10%	0.80%	1.10%	0.70%	1.50%	1%	6.10%	3.50%
Germany	0.90%	0.50%	1%	0.30%	1.10%	1.30%	2.40%	4%
Italy	1.40%	1.80%	2.20%	0.90%	3.10%	2%	4.90%	5.30%
Mexico	0.10%	0.10%	0.10%	0%	0.20%	0.10%	0%	0.20%
Netherlands	0.30%	0%	0.40%	0.50%	0.70%	0%	0.90%	1.20%
Slovenia	0%	0%	0.50%	0.50%	1.90%	0%	1.90%	2.10%
Spain	1.20%	0.40%	1.40%	0.90%	4%	2.70%	9.50%	11.30%
Sweden	0%	0%	0%	0.20%	0%	0.30%	3.40%	4.40%
Switzerland	0.20%	0.30%	0.20%	0%	0.30%	0.50%	0.70%	1.30%
United States	4.30%	1.50%	7.20%	2.30%	9.80%	4.90%	15.40%	20%

Table 27 Missing data: Difficulty with shopping

**Country**	**Men age 50-59**	**Women age 50-59**	**Men age 60-69**	**Women age 60-69**	**Men age 70-79**	**Women age 70-79**	**Men age 80 and over**	**Women age 80 and over**
United Kingdom	0%	0%	0%	0%	0%	0%	0%	0%
Austria	0%	0%	0%	0%	0%	0%	0%	0%
Belgium	0%	0%	0%	0%	0%	0%	0%	0%
China	0.10%	0.10%	0.10%	0.10%	0.10%	0.70%	0.40%	0.30%
Czech Republic	0%	0%	0%	0%	0%	0%	0%	0%
Denmark	0%	0%	0%	0%	0%	0%	0%	0%
Estonia	0%	0%	0%	0%	0%	0%	0%	0%
France	0%	0%	0%	0%	0%	0%	0%	0%
Germany	0%	0%	0%	0%	0%	0%	0%	0%
Italy	0%	0%	0%	0%	0%	0%	0%	0%
Mexico	1.20%	0.20%	2.10%	0.70%	3.30%	2.70%	7.30%	5.20%
Netherlands	0%	0%	0%	0%	0%	0%	0%	0%
Slovenia	0%	0%	0%	0%	0%	0%	0%	0%
Spain	0%	0%	0%	0%	0%	0%	0%	0%
Sweden	0%	0%	0%	0%	0%	0%	0%	0%
Switzerland	0%	0%	0%	0%	0%	0%	0%	0%
United States	0.10%	0%	0%	0%	0.10%	0%	0%	0.20%

Table 28 Missing data: Difficulty sitting for 2 hours

Country	Men age 50-59	Women age 50-59	Men age 60-69	Women age 60-69	Men age 70-79	Women age 70-79	Men age 80 and over	Women age 80 and over
United Kingdom	0%	0%	0%	0%	0%	0%	0%	0%
Austria	0%	0%	0%	0%	0%	0%	0%	0%
Belgium	0%	0%	0%	0%	0%	0%	0%	0%
China	0.60%	0.60%	0.30%	0.60%	0.60%	0.30%	0%	0%
Czech Republic	0%	0%	0%	0%	0%	0%	0%	0%
Denmark	0%	0%	0%	0%	0%	0%	0%	0%
Estonia	0%	0%	0%	0%	0%	0%	0%	0%
France	0%	0%	0%	0%	0%	0%	0%	0%
Germany	0%	0%	0%	0%	0%	0%	0%	0%
Italy	0%	0%	0%	0%	0%	0%	0%	0%
Mexico	0.10%	0.30%	0.30%	0.50%	0.50%	0.40%	0.60%	0.50%
Netherlands	0%	0%	0%	0%	0%	0%	0%	0%
Slovenia	0%	0%	0%	0%	0%	0%	0%	0%
Spain	0%	0%	0%	0%	0%	0%	0%	0%
Sweden	0%	0%	0%	0%	0%	0%	0%	0%
Switzerland	0%	0%	0%	0%	0%	0%	0%	0%
United States	0%	0.10%	0.10%	0%	0.10%	0.10%	0.20%	0.30%

Table 29 Missing data: Sleep problems

Country	Men age 50-59	Women age 50-59	Men age 60-69	Women age 60-69	Men age 70-79	Women age 70-79	Men age 80 and over	Women age 80 and over
United Kingdom	4.50%	2.80%	4.80%	2.80%	3.90%	2.70%	9.20%	12.20%
Austria	3.30%	1.60%	2.50%	0.90%	1.50%	1.10%	1.60%	1.30%
Belgium	0.70%	0.60%	0.30%	0.20%	0.40%	0.50%	4.40%	4.90%
China	12.10%	5.20%	6.80%	5.80%	6.50%	6.90%	17.70%	22.70%
Czech Republic	2.30%	1%	2.70%	1.30%	2.80%	1.60%	2.70%	4.30%
Denmark	1.40%	0.20%	0.60%	0%	3.10%	1%	4%	3.20%
Estonia	3.60%	0.90%	2.80%	0.40%	3.30%	0.60%	2.10%	1.90%
France	2.10%	1.10%	1.10%	0.60%	1.70%	0.90%	6.10%	3.30%
Germany	0.90%	0.50%	1%	0.30%	1.10%	0.90%	1.20%	5%
Italy	1.40%	1.80%	2.60%	0.90%	2.70%	2.20%	4.90%	4.80%
Mexico	0.10%	0.10%	0.20%	0%	0.10%	0%	0%	0.30%
Netherlands	0.30%	0%	0.20%	0.70%	0.70%	0%	0.90%	1.20%

Slovenia	0%	0%	0.50%	0%	1.50%	0.30%	1%	2.10%
Spain	0.70%	0.40%	1.20%	1.10%	4%	2.70%	11.60%	10.40%
Sweden	0%	0.70%	0%	0.20%	0%	0%	2.70%	5.60%
Switzerland	0.20%	0.30%	0%	0.20%	0.30%	0.20%	0.70%	1.30%
United States	4.30%	1.50%	7.20%	2.30%	9.90%	4.80%	15.50%	19.90%

Table 30 Missing data: Self-rated health

**Country**	**Men age 50-59**	**Women age 50-59**	**Men age 60-69**	**Women age 60-69**	**Men age 70-79**	**Women age 70-79**	**Men age 80 and over**	**Women age 80 and over**
United Kingdom	4.20%	2.40%	4.30%	2.40%	3.40%	2.20%	7.30%	11%
Austria	0%	0%	0%	0%	0%	0%	0%	0%
Belgium	0%	0%	0%	0%	0%	0%	0.40%	0.50%
China	14.70%	41.20%	16.20%	38.60%	19.90%	38.50%	16.90%	38.80%
Czech Republic	0%	0%	0%	0%	0.20%	0.20%	0%	0%
Denmark	0%	0%	0.30%	0%	0%	0%	0%	0%
Estonia	0.10%	0.20%	0.10%	0%	0.10%	0.20%	0%	0%
France	0%	0%	0.10%	0%	0.20%	0%	0%	0%
Germany	0%	0%	0%	0%	0%	0%	0%	0%
Italy	0%	0%	0%	0%	0%	0%	0%	0%
Mexico	0%	0%	0%	0%	0%	0%	0.20%	0%
Netherlands	0%	0%	0%	0%	0%	0%	0%	0%
Slovenia	0%	0%	0%	0%	0%	0%	0%	0%
Spain	0%	0%	0%	0%	0.20%	0%	0%	0.60%
Sweden	0%	0%	0%	0%	0%	0.30%	0%	1.90%
Switzerland	0%	0%	0%	0%	0%	0.20%	0%	0%
United States	0%	0%	0%	0%	0%	0.10%	0%	0.20%

Table 31 Missing data: Difficulty climbing stairs

**Country**	**Men age 50-59**	**Women age 50-59**	**Men age 60-69**	**Women age 60-69**	**Men age 70-79**	**Women age 70-79**	**Men age 80 and over**	**Women age 80 and over**
United Kingdom	0%	0%	0%	0%	0%	0%	0%	0%
Austria	0%	0%	0%	0%	0%	0%	0%	0%
Belgium	0%	0%	0%	0%	0%	0%	0%	0%
China	0.50%	0.60%	0.40%	0.90%	1.10%	0.70%	0.40%	1%
Czech Republic	0%	0%	0%	0%	0%	0%	0%	0%

Denmark	0%	0%	0%	0%	0%	0%	0%	0%
Estonia	0%	0%	0%	0%	0%	0%	0%	0%
France	0%	0%	0%	0%	0%	0%	0%	0%
Germany	0%	0%	0%	0%	0%	0%	0%	0%
Italy	0%	0%	0%	0%	0%	0%	0%	0%
Mexico	2.20%	3.70%	3.20%	5%	4.50%	7.50%	9.40%	11.60%
Netherlands	0%	0%	0%	0%	0%	0%	0%	0%
Slovenia	0%	0%	0%	0%	0%	0%	0%	0%
Spain	0%	0%	0%	0%	0%	0%	0%	0%
Sweden	0%	0%	0%	0%	0%	0%	0%	0%
Switzerland	0%	0%	0%	0%	0%	0%	0%	0%
United States	0%	0.10%	0.10%	0.10%	0.20%	0.30%	0.30%	0.30%

Table 32 Missing data: Difficulty stooping

**Country**	**Men age 50-59**	**Women age 50-59**	**Men age 60-69**	**Women age 60-69**	**Men age 70-79**	**Women age 70-79**	**Men age 80 and over**	**Women age 80 and over**
United Kingdom	0%	0%	0%	0%	0%	0%	0%	0%
Austria	0%	0%	0%	0%	0%	0%	0%	0%
Belgium	0%	0%	0%	0%	0%	0%	0%	0%
China	0.10%	0.10%	0.10%	0.10%	0%	0.40%	0%	0%
Czech Republic	0%	0%	0%	0%	0%	0%	0%	0%
Denmark	0%	0%	0%	0%	0%	0%	0%	0%
Estonia	0%	0%	0%	0%	0%	0%	0%	0%
France	0%	0%	0%	0%	0%	0%	0%	0%
Germany	0%	0%	0%	0%	0%	0%	0%	0%
Italy	0%	0%	0%	0%	0%	0%	0%	0%
Mexico	0.10%	0.30%	0.20%	0.70%	0.80%	1.10%	1.80%	2.10%
Netherlands	0%	0%	0%	0%	0%	0%	0%	0%
Slovenia	0%	0%	0%	0%	0%	0%	0%	0%
Spain	0%	0%	0%	0%	0%	0%	0%	0%
Sweden	0%	0%	0%	0%	0%	0%	0%	0%
Switzerland	0%	0%	0%	0%	0%	0%	0%	0%
United States	0.10%	0.20%	0%	0.10%	0.10%	0.20%	0.20%	0.40%

Table 33 Missing data: Stroke

**Country**	**Men age 50-59**	**Women age 50-59**	**Men age 60-69**	**Women age 60-69**	**Men age 70-79**	**Women age 70-79**	**Men age 80 and over**	**Women age 80 and over**

United Kingdom	0%	0%	0%	0%	0%	0%	0%	0%
Austria	0%	0%	0%	0%	0%	0%	0%	0%
Belgium	0%	0%	0%	0%	0%	0%	0%	0%
China	0.40%	0.10%	0.20%	0.10%	0.30%	0.30%	0%	0.30%
Czech Republic	0%	0%	0%	0%	0%	0%	0.50%	0%
Denmark	0%	0%	0%	0%	0%	0%	0%	0%
Estonia	0%	0%	0%	0%	0%	0%	0%	0%
France	0%	0%	0%	0%	0%	0%	0%	0%
Germany	0%	0%	0%	0%	0%	0%	0%	0%
Italy	0%	0%	0%	0%	0%	0%	0%	0%
Mexico	0.20%	0%	0.10%	0%	0%	0%	0%	0.20%
Netherlands	0%	0%	0%	0%	0%	0%	0%	0%
Slovenia	0%	0%	0%	0%	0%	0%	0%	0%
Spain	0%	0%	0%	0%	0%	0%	0%	0%
Sweden	0%	0%	0%	0%	0%	0%	0%	0%
Switzerland	0%	0%	0%	0%	0%	0%	0%	0%
United States	0%	0%	0.10%	0%	0.10%	0.10%	0.10%	0.20%

Table 34 Missing data: Difficulty toileting

**Country**	**Men age 50-59**	**Women age 50-59**	**Men age 60-69**	**Women age 60-69**	**Men age 70-79**	**Women age 70-79**	**Men age 80 and over**	**Women age 80 and over**
United Kingdom	0%	0%	0%	0%	0%	0%	0%	0%
Austria	0%	0%	0%	0%	0%	0%	0%	0%
Belgium	0%	0%	0%	0%	0%	0%	0%	0%
China	0.70%	0.60%	0.30%	0.60%	0.60%	0.20%	0%	0%
Czech Republic	0%	0%	0%	0%	0%	0%	0%	0%
Denmark	0%	0%	0%	0%	0%	0%	0%	0%
Estonia	0%	0%	0%	0%	0%	0%	0%	0%
France	0%	0%	0%	0%	0%	0%	0%	0%
Germany	0%	0%	0%	0%	0%	0%	0%	0%
Italy	0%	0%	0%	0%	0%	0%	0%	0%
Mexico	0%	0.20%	0.40%	0.20%	0.20%	0.30%	1%	0.20%
Netherlands	0%	0%	0%	0%	0%	0%	0%	0%
Slovenia	0%	0%	0%	0%	0%	0%	0%	0%
Spain	0%	0%	0%	0%	0%	0%	0%	0%
Sweden	0%	0%	0%	0%	0%	0%	0%	0%
Switzerland	0%	0%	0%	0%	0%	0%	0%	0%
United States	0%	0%	0%	0.10%	0.10%	0%	0.30%	0.20%

Table 35 Missing data: Visual problems

Country	Men age 50-59	Women age 50-59	Men age 60-69	Women age 60-69	Men age 70-79	Women age 70-79	Men age 80 and over	Women age 80 and over
United Kingdom	0%	0%	0%	0%	0%	0%	0%	0%
Austria	0%	0%	0.30%	0.20%	0.20%	0.30%	0%	0.30%
Belgium	0%	0.10%	0%	0%	0.20%	0%	0%	0%
China	0%	0%	0%	0%	0.10%	0%	0.40%	0%
Czech Republic	0.50%	0.30%	0%	0.20%	0.60%	0.30%	0%	0%
Denmark	0%	0%	0%	0%	0%	0%	0%	0%
Estonia	0.10%	0%	0%	0%	0%	0%	0%	0%
France	0%	0%	0%	0%	0%	0%	0%	0%
Germany	0.90%	0.50%	0%	0%	0%	0%	0%	0%
Italy	0.80%	1.20%	0.70%	0.70%	0.60%	0.60%	0%	0%
Mexico	0%	0%	0%	0%	0%	0%	0%	0%
Netherlands	0%	0%	0%	0%	0%	0%	0%	0%
Slovenia	0%	0%	0%	0%	0.40%	0%	0%	0%
Spain	0%	0.20%	0%	0%	0%	0%	0%	0%
Sweden	0%	0%	0%	0%	0%	0%	0%	0%
Switzerland	0.20%	0.20%	0%	0%	0%	0%	0%	0%
United States	0.10%	0.10%	0.30%	0.10%	0.30%	0.30%	0.40%	0.40%

Table 36 Missing data: Difficulty walking 100 metres

Country	Men age 50-59	Women age 50-59	Men age 60-69	Women age 60-69	Men age 70-79	Women age 70-79	Men age 80 and over	Women age 80 and over
United Kingdom	0%	0%	0%	0%	0%	0%	0%	0%
Austria	0%	0%	0%	0%	0%	0%	0%	0%
Belgium	0%	0%	0%	0%	0%	0%	0%	0%
China	2.90%	4.90%	5.40%	9.20%	13.50%	22.40%	29.30%	44.50%
Czech Republic	0%	0%	0%	0%	0%	0%	0%	0%
Denmark	0%	0%	0%	0%	0%	0%	0%	0%
Estonia	0%	0%	0%	0%	0%	0%	0%	0%
France	0%	0%	0%	0%	0%	0%	0%	0%
Germany	0%	0%	0%	0%	0%	0%	0%	0%
Italy	0%	0%	0%	0%	0%	0%	0%	0%

Mexico	0.10%	0.20%	0%	0.10%	0.20%	0.60%	0.80%	2.30%
Netherlands	0%	0%	0%	0%	0%	0%	0%	0%
Slovenia	0%	0%	0%	0%	0%	0%	0%	0%
Spain	0%	0%	0%	0%	0%	0%	0%	0%
Sweden	0%	0%	0%	0%	0%	0%	0%	0%
Switzerland	0%	0%	0%	0%	0%	0%	0%	0%
United States	0%	0.10%	0%	0.10%	0%	0%	0.10%	0.10%

Figure 16 Median (interquartile range) frailty index for each country after excluding each deficit from the frailty index in turn

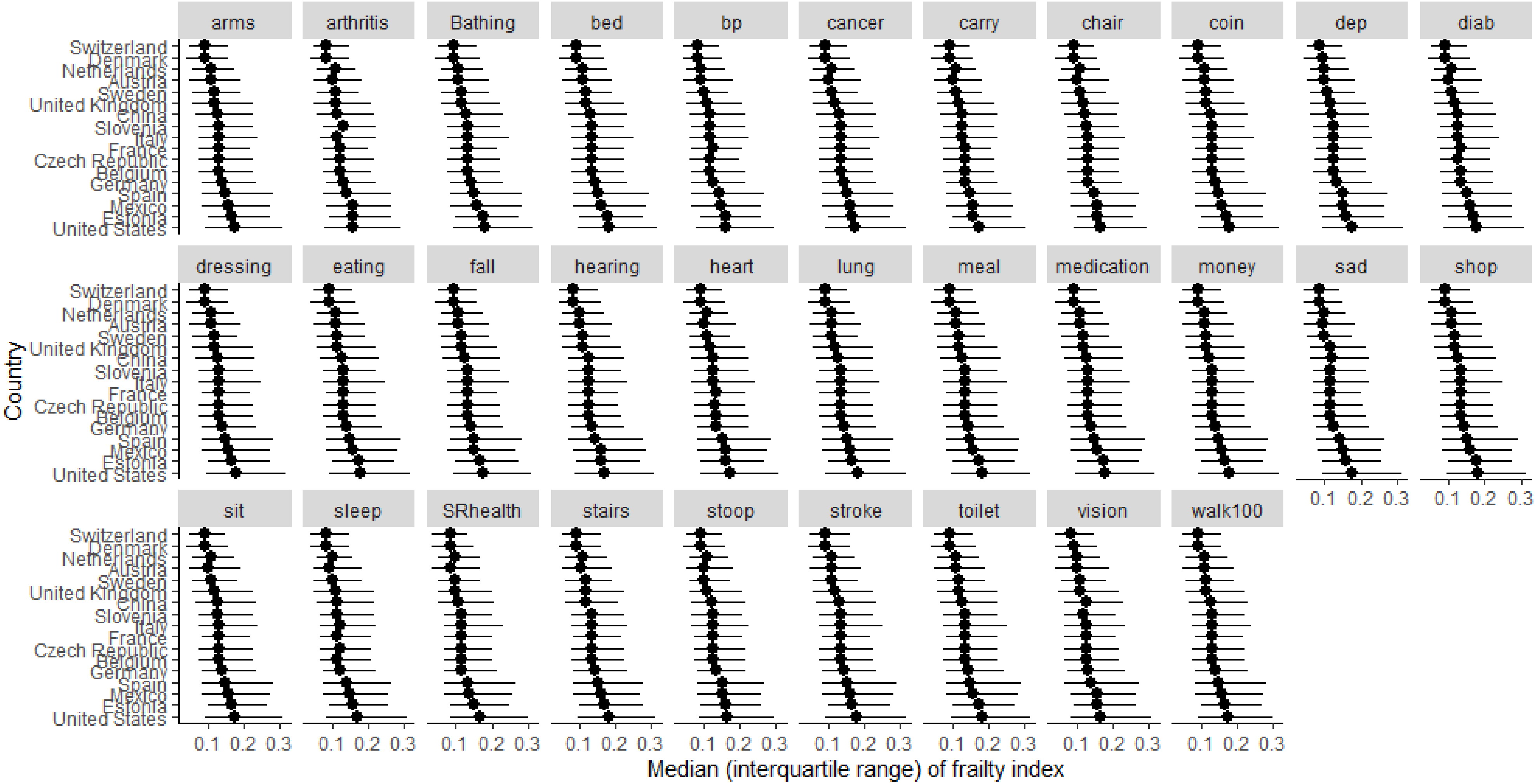

Figure 17 Mortality analysis - 2-year follow-up - zero-state population - after excluding each deficit in turn

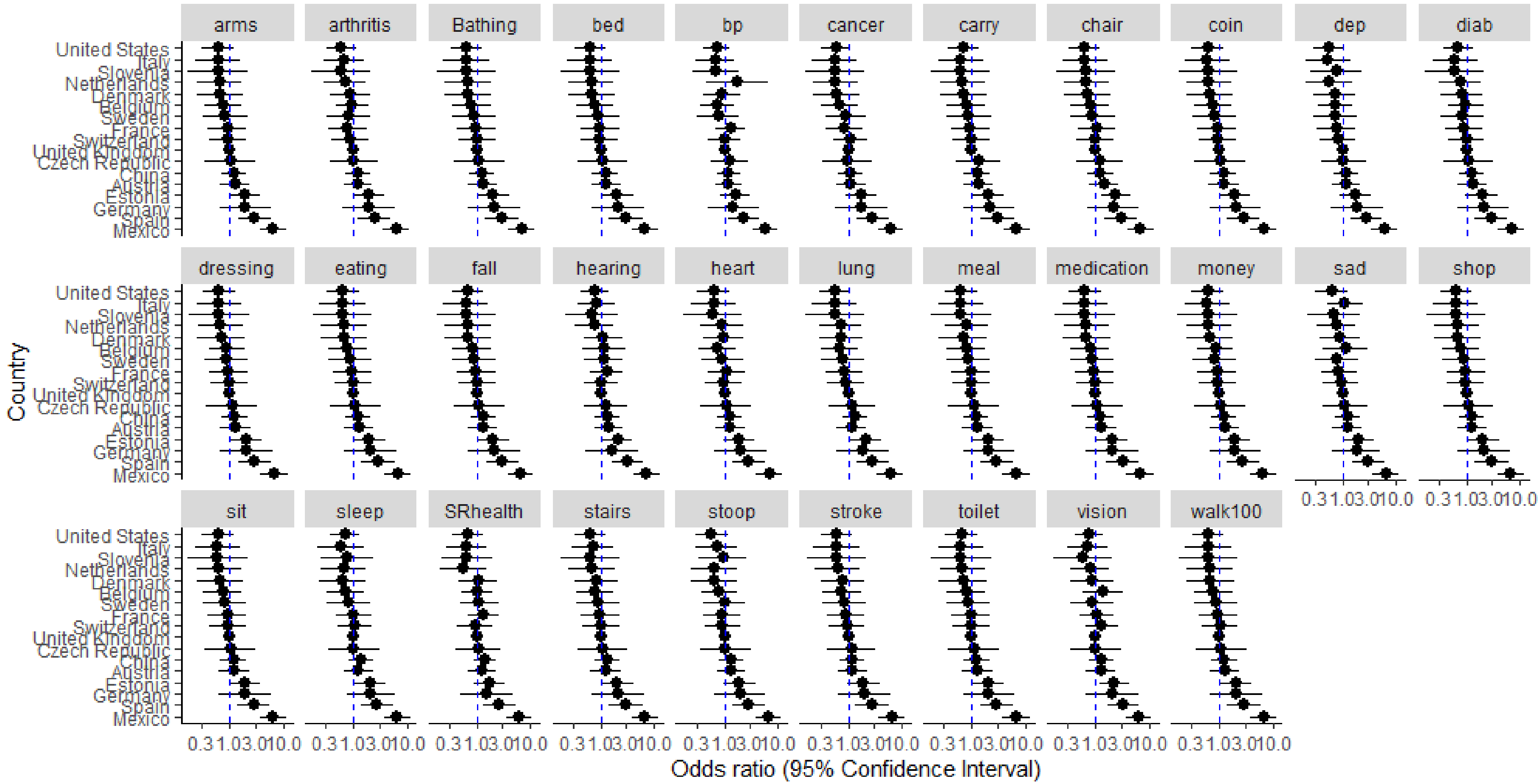

Figure 18 Mortality analysis - 2-year follow-up - non-zero-state population - after excluding each deficit in turn

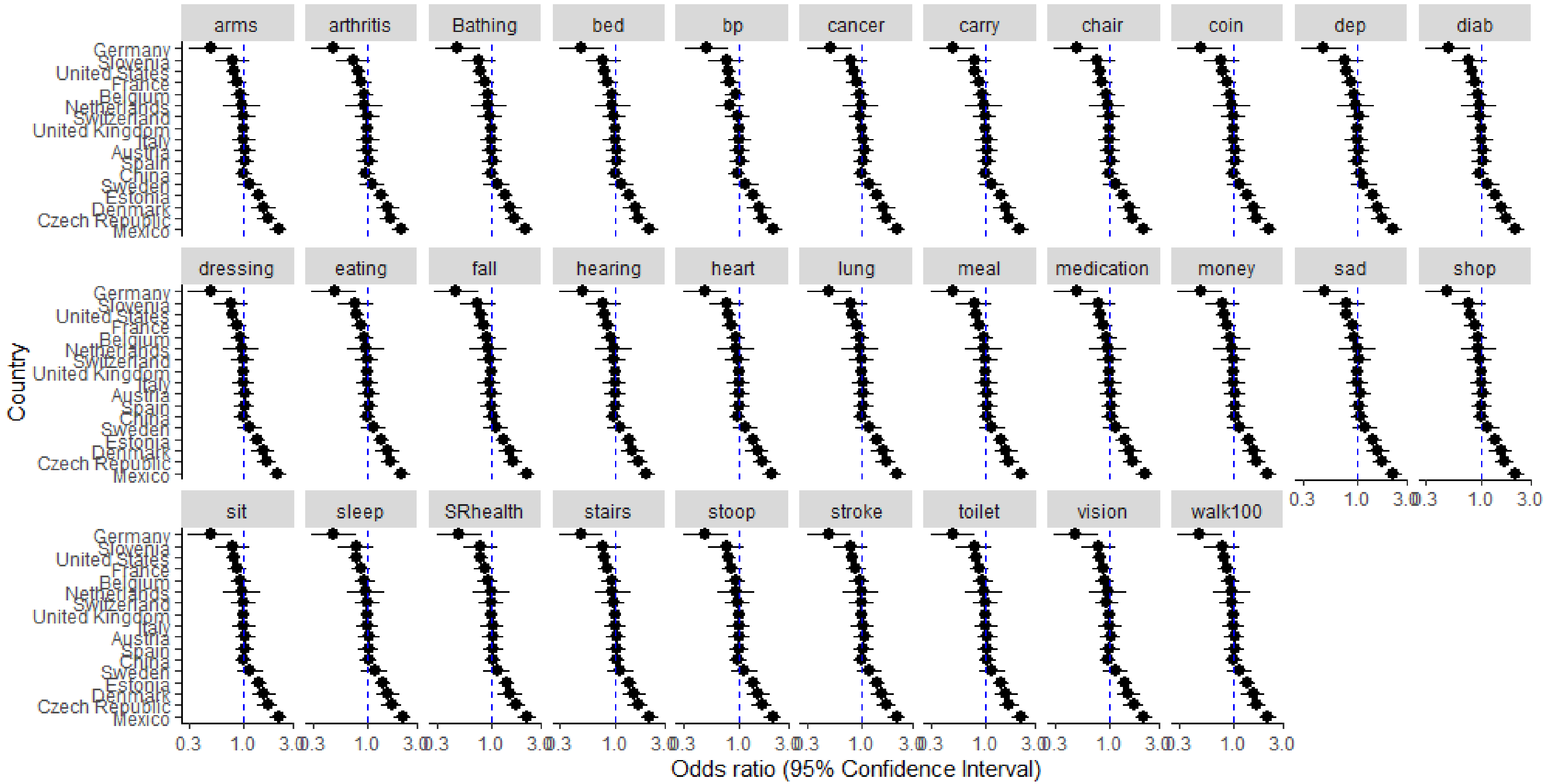

Figure 19 Deficit accumulation analysis - 2-year follow-up - zero-state population - after excluding each deficit in turn

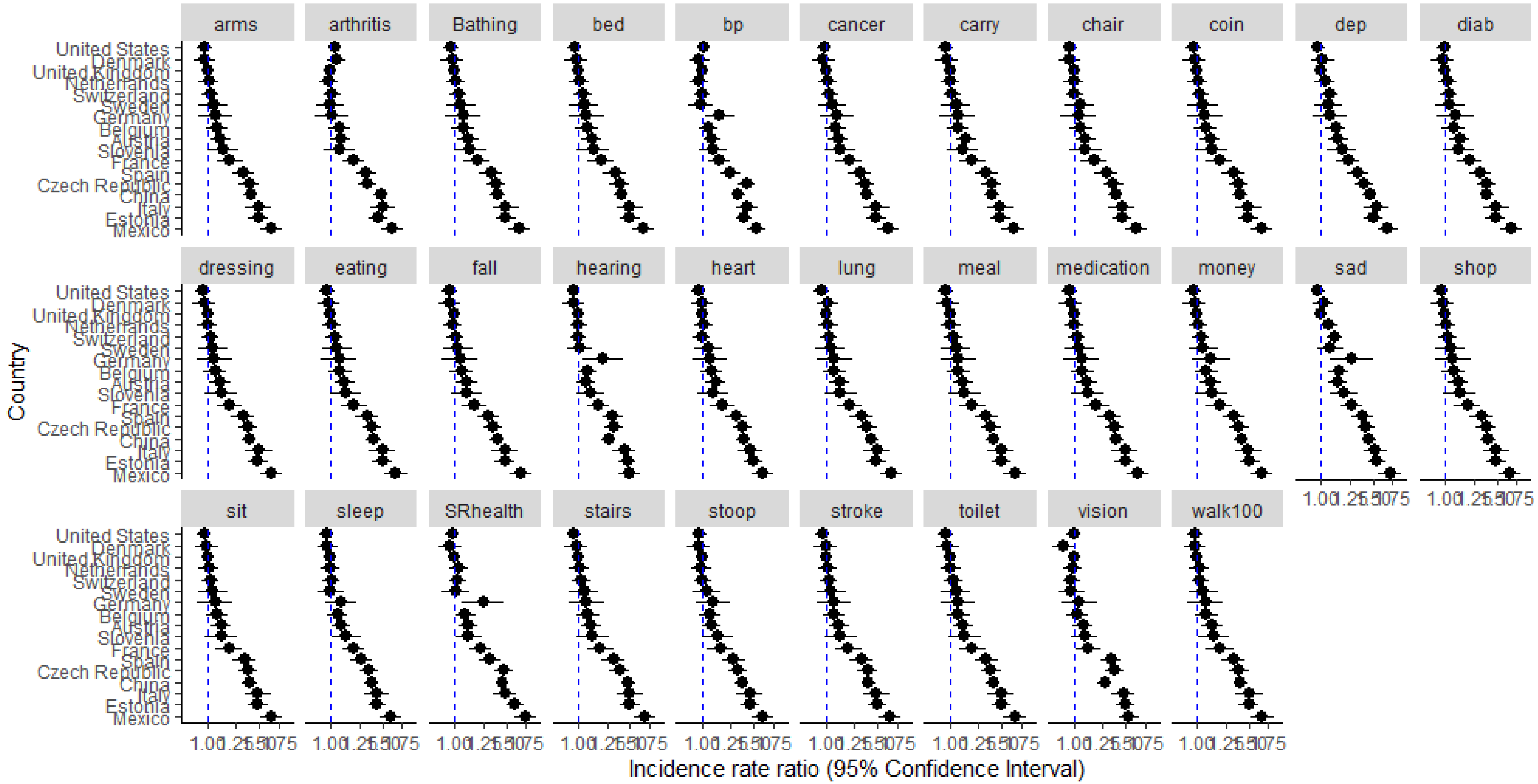

Figure 20 Deficit accumulation analysis - 2-year follow-up - non-zero-state population - after excluding each deficit in turn

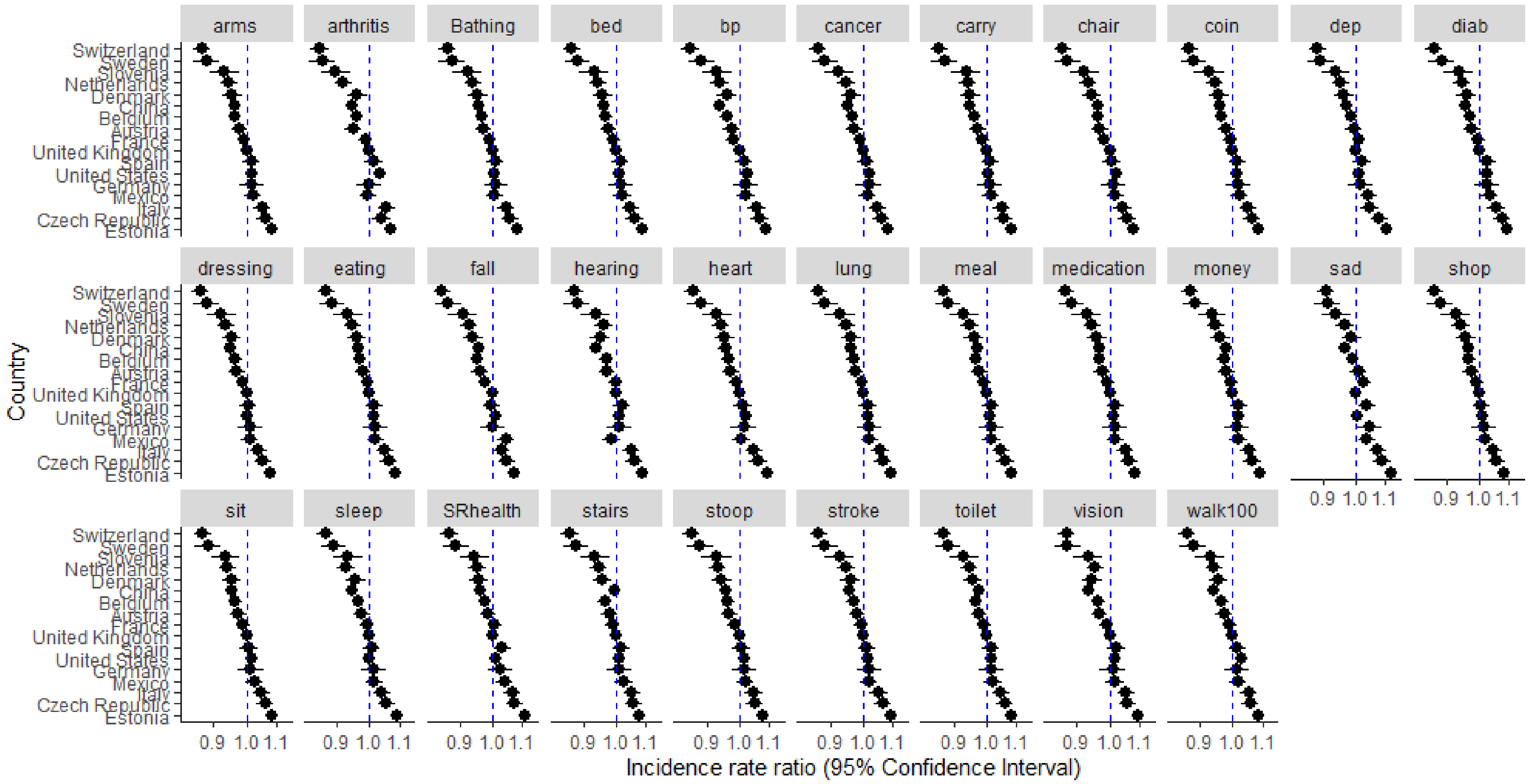

Supplement: Supplementary file 1 [file supplementary_materials_afag242.pdf]
